# Comparative transcriptomics of stem rust resistance in wheat NILs mediated by *Sr24* rust resistance gene

**DOI:** 10.1371/journal.pone.0295202

**Published:** 2023-12-11

**Authors:** Gautam Vishwakarma, Ajay Saini, Subhash Chander Bhardwaj, Satish Kumar, Bikram Kishore Das

**Affiliations:** 1 Nuclear Agriculture and Biotechnology Division, Bhabha Atomic Research Centre, Trombay, Mumbai, Maharashtra, India; 2 Homi Bhabha National Institute, Mumbai, India; 3 Molecular Biology Division, Bhabha Atomic Research Centre, Trombay, Mumbai, Maharashtra, India; 4 ICAR—Indian Institute of Wheat and Barley Research, Shimla, India; 5 ICAR—Indian Institute of Wheat and Barley Research, Karnal, Haryana, India; Institute of Genetics and Developmental Biology Chinese Academy of Sciences, CHINA

## Abstract

Stem rust of wheat is a deleterious fungal disease across the globe causing severe yield losses. Although, many stem rust resistance genes (*Sr*) are being used in wheat breeding programs, new emerging stem rust pathotypes are a challenge to important *Sr* genes. In recent years, multiple studies on leaf and yellow rust molecular mechanism have been done, however, for stem rust such studies are lacking. Current study investigated stem rust induced response in the susceptible wheat genotype C306 and its Near Isogenic Line (NIL) for *Sr24* gene, HW2004, using microarray analysis to understand the transcriptomic differences at different stages of infection. Results showed that HW2004 has higher basal levels of several important genes involved in pathogen detection, defence, and display early activation of multiple defence mechanisms. Further Gene Ontology (GO) and pathway analysis identified important genes responsible for pathogen detection, downstream signalling cascades and transcription factors (TFs) involved in activation and mediation of defence responses. Results suggest that generation of Reactive Oxygen Species (ROS), cytoskeletal rearrangement, activation of multiple hydrolases, and lipid metabolism mediated biosynthesis of certain secondary metabolites are collectively involved in *Sr24-*mediated defence in HW2004, in response to stem rust infection. Novel and unannotated, but highly responsive genes were also identified, which may also contribute towards resistance phenotype. Furthermore, certain DEGs also mapped close to the *Sr24-*linked marker on *Thinopyrum elongatum* translocated fragment on wheat 3E chromosome, which advocate further investigations for better insights of the *Sr24-*mediated stem rust resistance.

## Introduction

Wheat is the third most important cereal crop globally, and crucial for economic as well as nutritional security world-wide [1]. Wheat production is affected by numerous biotic (rusts, smuts, powdery mildew and fusarium head blight) and abiotic (heat, drought, shorter season duration, salt and alkalinity) stresses [2]. Among the biotic stresses, rusts of wheat are important threat to global wheat production [3]. Due to rapidly evolving virulent pathotypes and large area of impact, rust diseases have become a major challenge for wheat breeders [4]. Stem rust of wheat a highly deleterious disease (caused by *Puccinia graminis* f. sp. *tritici* (*Pgt*)), is estimated to cause up to 50% loss in yield or even higher if infection starts at an early stage [3]. Effective management of stem and other rusts involve employment of resistance genes (*R*) and chemical intervention [2]. Close to 60 *Sr* loci are now reported, including race specific all duration resistance genes as well as broad-spectrum adult plant resistance (APR) genes [5]. Although chemical intervention is still utilized to control wheat rusts, genetic control of the disease using ‘*R*’ genes is still the most economical and environment sustainable approach [3, 6].

Resistance mediated by ‘*R*’ genes is based on its successful interaction with corresponding Avirulence (*Avr*) factors of pathogen (incompatible reaction). Failure to recognize the *Avr* factors lead to susceptibility and disease development (compatible reaction) [7]. Plant defence mechanisms against pathogens is based on immune responses operating at two-levels [5, 8]. The primary/basal defence mechanism directed against a large number of pathogens involves PRR-mediated recognition of conserved PAMP, and is termed as PAMP Triggered Immunity (PTI) [9]. Here, the transmembrane PRR protein having LRR (extracellular) and kinase (intracellular) domain binds to pathogen-specific signatures, activating defence mechanisms orchestrated by PR proteins, ROS modulation and cell wall strengthening [9, 10]. Plant pathogens can evade the detection by the PRRs by secreting the effector molecules directly inside the host cell, using specialized secretion system [8]. However, plants use ‘*R*’ gene to detect these effector molecules and activate second level of defence response, termed as Effector Triggered Immunity (ETI), which typically results in hypersensitivity response (HR) [11, 12]. The ‘*R’* proteins are classified into five groups, of which the NBS-LRR (NB-ARC-LRR) group is the largest [13]. In ETI interaction between effector and ‘*R*’ gene receptors may be direct, or it may detect the modifications on the host cell surface, leading to a relatively rapid defence response than PTI [11, 14].

Multiple ‘*R*’ genes have been cloned, however, the mechanisms responsible for conferring resistance are not understood completely [15]. Reports on wheat leaf and yellow rust have provided insights into important candidate genes and pathways involved in resistance mediated by both race-specific and APR genes, particularly on, resistance mechanisms in mixed races (field inoculum) [16–19]. Emergence of new virulence *Pgt* pathotypes (e.g. Ug99 group), has resulted in breakdown of several important ‘*Sr*’ genes viz. *Sr31* and *Sr24*, which is alarming for breeders and pathologists [3–5]. Hence, detailed studies are needed for understanding molecular basis of resistance conferred by different ‘*R*’ genes against stem rust disease in wheat. Unlike leaf and yellow rust, global analysis of resistance response is lacking in case of wheat stem rust. *Sr24* (linked to *Lr24*, chromosome: 3D), an important *Sr* gene present in global wheat varieties, confers resistance to most of stem rust races (includes Ug99 race TTKSK, all races in India except 62G29-1) [20–22]. Being originated from alien source *Thinopyrum elongatum*, its molecular understanding is not well known, however, due to presence of tightly linked DNA markers (e.g. *Xbarc71*, *Sr24#12*) it has been widely deployed in many wheat varieties across the globe [22, 23]. The wild relative of wheat *T*. *elongatum*, (referred to as tall wheat grass, E genome) has been utilized for transferring multiple resistance genes to wheat, including *Sr24* [23, 24]. Gene transfer using wide hybridization from *T*. *elongatum* resulted in translocation stock lines at 3D, 3DL, 3BL and 1BS [25–28]. Further, the 3D/Ag lines developed by Sear’s were used for developing white seed wheat varieties in Australia and was then used as source for breeding Indian varieties [29].

In this study, NILs for ‘*Sr24*’ were used for understanding transcriptomic differences between resistance and susceptibility upon challenge with a local stem rust race 7G11. Infection with 7G11, lead to highly susceptible reaction on recurrent parent C306 and resistant type reaction on HW2004. High-throughput microarray analysis revealed peak transcriptional difference at early stages of infection, with upregulation of genes for pathogen detection and defence mechanism activation. Overall, genes for receptors, activation of signalling cascade, TFs for defence genes, and defence responses including ROS, secondary metabolites, and PR proteins showed upregulation in resistant NIL (HW2004) at early stage of infection. Certain Differentially Expressed Genes (DEGs) were also mapped on to the *T*. *elongatum* translocated fragment, and in vicinity to the *Sr24*-linked marker, including both the uncharacterized as well as disease responsive genes.

## Materials and methods

### Plant material and rust inoculation

Wheat stem rust susceptible variety C306 (lacking *Sr24* gene) and its NIL genotype HW2004 with *Sr24* (also known as Unnath C306; C306 + *Sr24*), were obtained from Indian Agricultural Research Institute Delhi. The plants were grown in an MLR-351H plant growth chamber (SANYO, Japan) under following conditions: 16 hours light/ 8 hours dark cycle, temperature: 25°C (light) 18°C (dark) and humidity: 80–95%. *Puccinia graminis* f. sp. *tritici* (*Pgt*) pathotype 7G11 spores were received from IIWBR Regional Station, Flowerdale, Shimla, (infection type 1 on HW2004, 4 on C306) these were multiplied and maintained on C306. Wheat seedlings (at GS13, Zadok scale) were inoculated with stem rust spore suspension (concentration: ~ 6x10^5^ spores ml^-1^ in water containing 1 ppm Tween 20 detergent) as described by Bhardwaj 2011 [30, 31]. Stem rust infection phenotyping was done after 14 days post inoculation (dpi). Leaf tissue samples were collected at three time-points i.e., 0h, 10h and 72h post inoculation (hpi), snap freezed in liquid nitrogen, and stored at -80°C till further use. For each time-point tissue samples of three plants were pooled together, and experiment was repeated after one month for the second independent biological replicate.

### Microarray analysis

Total RNA was extracted from leaf tissue (500 mg) using TRIzol (Life Technologies, USA) method, and treated with DNase I enzyme (Invitrogen, USA) as per manufacturer’s instructions. Quantification of RNA preparations was done on Nanodrop 1000 Spectrophotometer (Thermo Fisher Scientific, USA) and integrity was assessed on 2100 Bioanalyzer (Agilent Technologies, USA). Good quality RNA samples (OD 260/280 values: 1.8–2.2, 28S/18S rRNA ratio: 2:1, and RNA integrity number (RIN) of >7) were used for microarray analysis, carried out at Genotypic Technologies Pvt Ltd. (Bangalore, India). Total RNA was labelled using Agilent Quick Amp labelling kit (part number: 5190–0442) as per the recommended protocol. Briefly, total RNA (500 ng) was reverse transcribed using oligo (dT) primer containing T7 promoter sequence, converted into double-stranded cDNA, and used for generation of Cy3-labelled complementary RNA (cRNA) by T7 RNA polymerase. The Cy3-labelled cRNAs were cleaned using Qiagen RNeasy columns (Qiagen, Germany, Cat No. 74106). 1650 ng of Cy3-labelled cRNA was hybridized on an Agilent wheat 4x44K microarray (AMADID 22297, Agilent Technologies, USA) using Agilent Gene Expression Hybridization Kit (Part No. 5190–0404), and inside Agilent Sure hybridization chamber for 16 hours at 65°C. Microarray slides were washed using Agilent Gene Expression wash buffer (Part No. 5188–5327) and scanned on G2600D microarray scanner (Agilent technologies, USA). Extraction of data from microarray slide images was done using Agilent Feature Extraction software Ver-11.5 and analysed by Agilent GeneSpring GX Version 12 (GS) software. Briefly, the signals were corrected for background and baseline transformed to the median of all spots, intra- and inter-microarray normalization was done for all the samples. Principal Component Analysis (PCA) was done to assess quality of independent replicates, and global normalization (normalized by 75^th^ percentile shift method) of spot intensities was done using GS software (S1 Fig). Spot intensities were log_2_ transformed and averaged for two replicate spots. DEGs were identified using unpaired student t-test having a Fold Change (FC) value ≥ 2.0 with Benjamini-Hochberg FDR corrected *p*-value ≤ 0.05, and visualized in volcano plots (S2 Fig) [32]. Expression profiles of DEGs were analyzed by hierarchical clustering complete linkage method using Heatmap Illustrator Ver 2.0 [33]. The microarray data files have been submitted to the Gene Expression Omnibus (GEO) database (accession no GSE207175).

### Functional annotation of DEGs

Transcript consensus (TC) sequences specific to the microarray probes were retrieved from *Triticum aestivum* gene indices TAGI 12 release of JCVI/TIGR plant gene indices [34]. TC sequences were used for similarity search analysis against the wheat RefSeq v2.0 (genotype: Chinese Spring) standard and used for further analysis [35]. Biological functions were assigned using Gene Ontology (GO) annotation for Molecular Function (MF), Biological Process (BP) and Cellular Component (CC) using quickGO [36]. Enrichment and visualization of GO terms was done using ShinyGO tool [37]. Important pathways affected by these DEGs were identified using KEGG KOLA and DAVID database [38–40].

### Validation of differential gene expression by RT-qPCR analysis

10μg of isolated total RNA was reverse transcribed using Superscript III Reverse transcriptase (Life Technologies, USA) and Oligo (dT)_20_ primer (Life Technologies, USA), as per the recommended protocol. Gene-specific primers were designed using Primer3 software and evaluated for specificity with NCBI Primer BLAST (S1 Table) [41]. Real time PCR assays were carried out on Eppendorf realplex 4 thermal cycler (Eppendorf, Germany) using Sigma SYBR Green JumpStart *Taq* Ready Mix (Sigma Aldrich, USA) as per manufacturer’s instruction. Data analysis was done using Eppendorf realplex software Ver2.2. Wheat *Actin* gene (*TaAct*) was used as reference gene for normalizing the expression of each gene and FC was calculated using 2^-ΔΔCt^ method [42]. The RT-qPCR analysis was repeated three times with independent biological replicates.

### Mapping of stem rust responsive DEGs to the *Thinopyrum elongatum* genome

The stem rust responsive DEGs were mapped to the *T*. *elongatum* chromosomal region harbouring the *Sr24*-linked marker using the following approach. Chromosome 3E of *T*. *elongatum* genomic sequence (NCBI genomes reference genome number ASM1179987v1), was used for mapping of *Sr24* tightly linked SSR marker *Xbarc71* (NCBI GenBank id BV211796.1) and stem rust responsive DEGs using NCBI-BLAST tool. The mapped DEGs were filtered to 70Mbp region from end terminal of 3E chromosome (equivalent to approximately ±2 cM from *Xbarc71* SSR marker). The mapped gene coordinates were visualized using ChromoMap Ver 4.1.1 [43].

## Results and discussion

### Wheat NILs phenotypic reaction to stem rust pathogen

**Fig 1:**
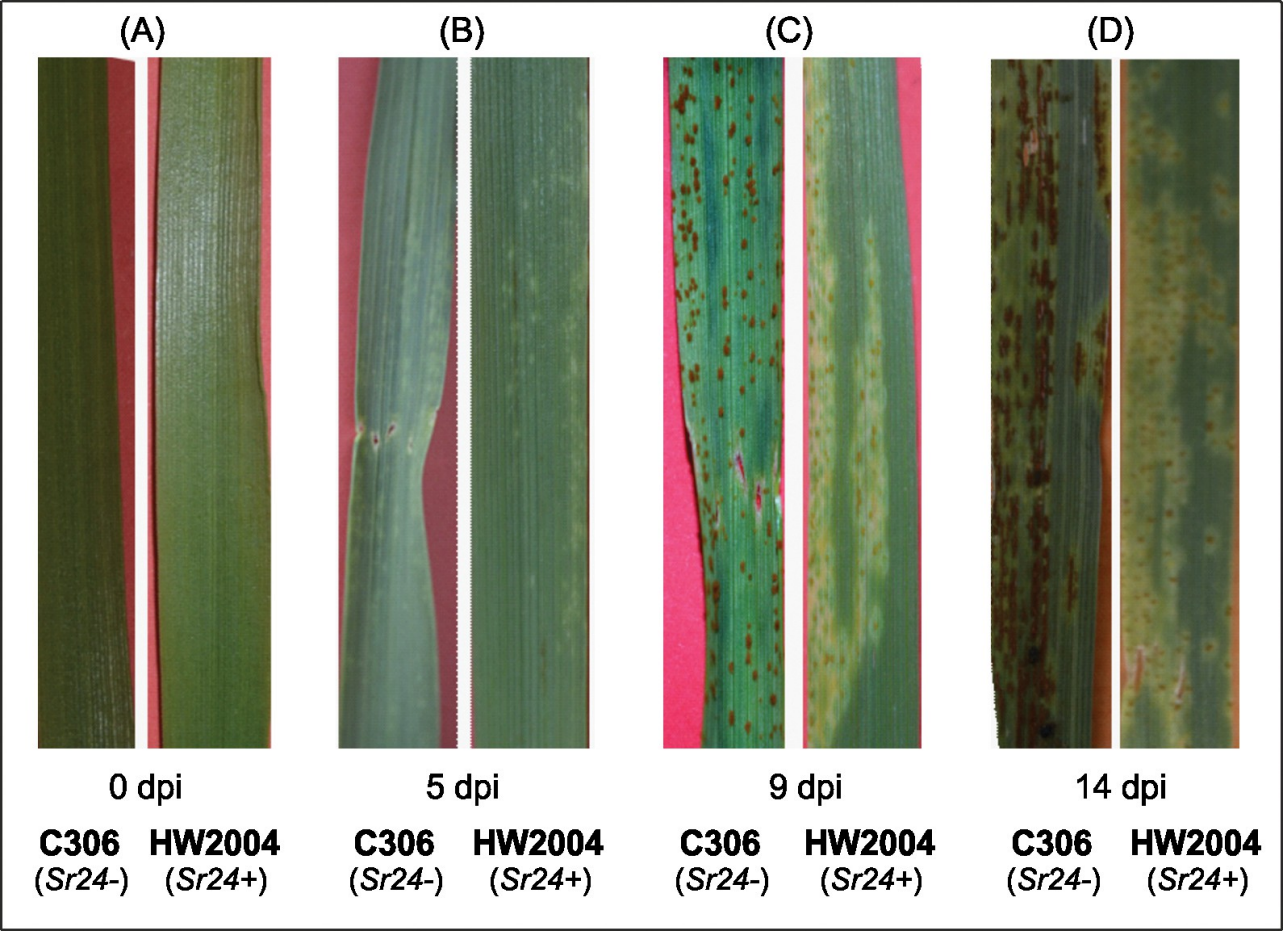
Phenotypic expression of stem rust of wheat. Comparison of stem rust infection in susceptible (C306, *Sr24*^-^) and resistant (HW2004, *Sr24*^+^) wheat near isogenic lines (NILs) differing in *Sr24* stem rust resistance gene at multiple time-points up to14 dpi (days post inoculation): (A) 0 dpi, (B) 5 dpi, (C) 9 dpi, (D) 14 dpi.

The wheat NILs (C306, susceptible and HW2004, resistant) differing in *Sr24* stem rust resistance gene showed differential rust reaction post inoculation of the pathogen, *Pgt* pathotype 7G11. Inoculated plants started showing visible response to stem rust infection 5 dpi, where HW2004 showed reduced yellow flecks compared to C306 (Fig 1A and 1B). At 9 dpi brick red coloured fungal spore pustules or uredospores started to appear in flecked regions in C306, whereas the HW2004 showed minute uredospores surrounded by large flecks, indicative of hypersensitive immune response against the pathogen (Fig 1C). At 14 dpi, uredospores showed profuse growth on the leaf surface of C306, compared to HW2004, where it was largely restricted (Fig 1D). Appearance of HR symptoms are typical of resistance response to fungal pathogen in plants, and have also been reported in wheat leaf as well as stripe rust infections [16, 19].

### Wheat NILs exhibit transcriptomic differences in response to stem rust

**Fig 2:**
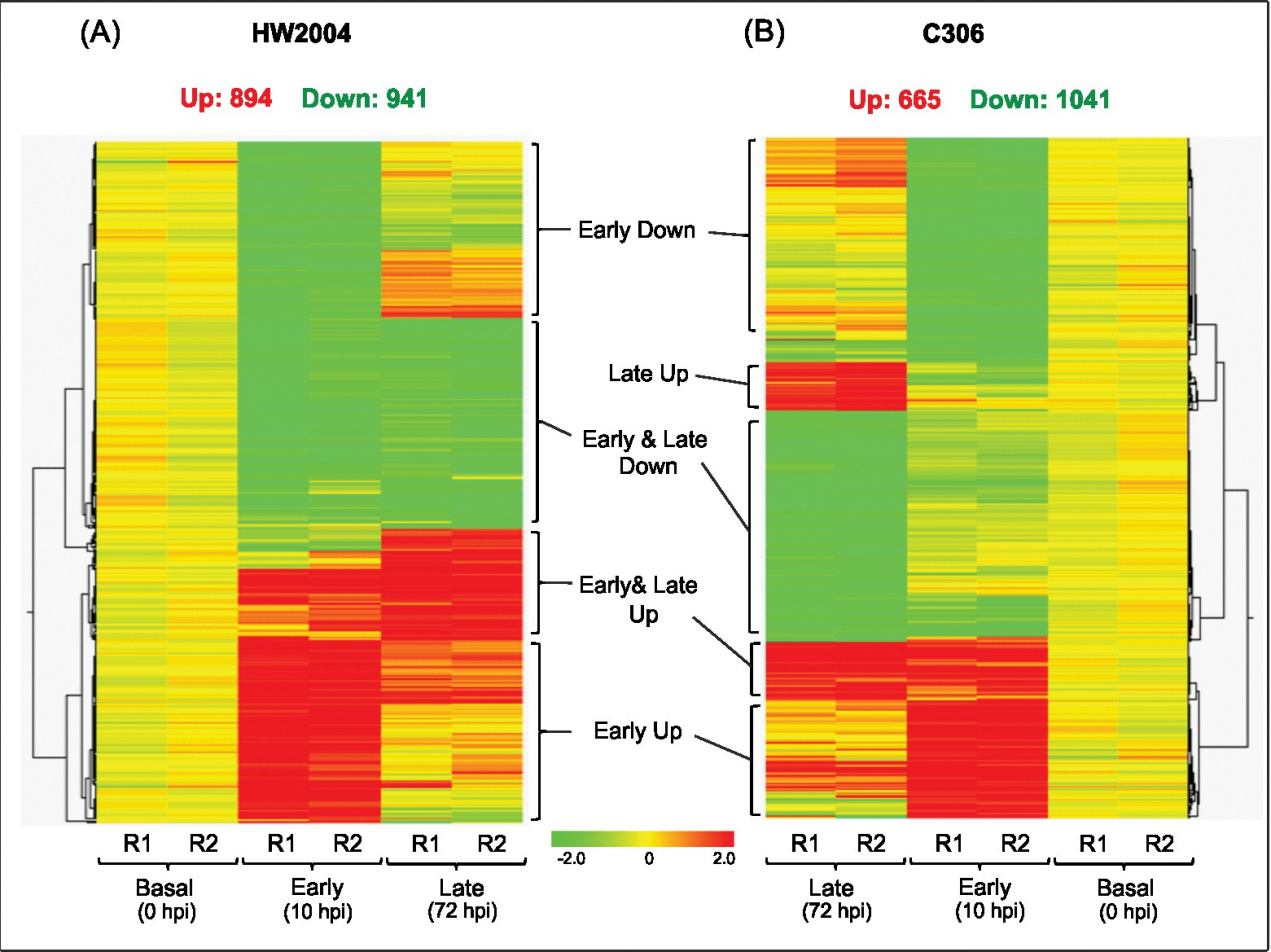
Global expression profile of DEGs in response to *Pgt* infection. Overview of transcriptomic response at three time-points (0 hpi, 10 hpi and 72 hpi) in (A) HW2004 and (B) C306 based on hierarchal clustering. Up refers to upregulated and Down to downregulated DEGs in the respective NILs, R1 & R2 refer to the two independent biological replicates.

The current study employed NILs for stem rust resistance gene *Sr24*, developed after seven backcrosses thus minimizing the background transcriptomic differences [44]. Previous studies on wheat leaf and stripe rust have reported activation of various defence response within few hours of pathogen inoculation [17, 18]. In the current study the transcriptomic profiles were studied at basal level (0 hpi), and to capture early as well as late events after infection (early: 10 hpi and late: 72 hpi), and DEGs at these stages were identified (S3 Fig, S2–S4 Tables). Expression profiles showed higher number of DEGs in HW2004 (894 genes, including higher number of upregulated DEGs) compared to C306 (665 genes) indicating differential transcriptomic reprogramming upon *Pgt* infection (Fig 2, S4 Fig, S2 and S3 Tables).

**Fig 3:**
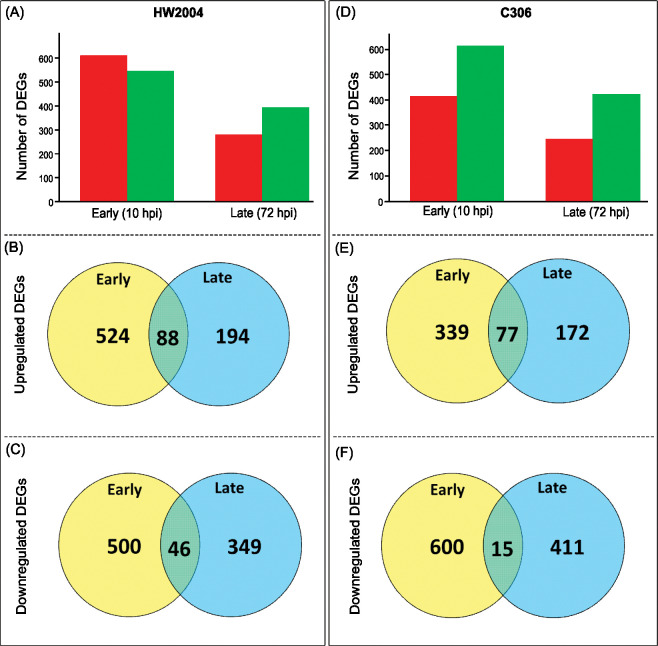
Number of DEGs at early (10 hpi) and late (72 hpi) stages of infection in the two NILS. (A) Number of DEGs at 10 hpi and 72 hpi compared to 0 hpi time-point in HW2004. (B) Venn diagram representation of common and unique upregulated DEGs at 10 hpi and 72 hpi in HW2004. (C) Common and unique downregulated DEGs at 10 hpi and 72 hpi in HW2004. (D) Number of DEGs at 10 hpi and 72 hpi compared to 0 hpi time-point in C306. (E) Venn diagram representation of common and unique upregulated DEGs at 10 hpi and 72 hpi in C306. (F) Common and unique downregulated DEGs at 10 hpi and 72 hpi in C306. Numbers in the Venn diagram represent number of DEGs in the respective stage of infection.

In HW2004 at early stage (10 hpi), 612 genes were found to be upregulated (524 uniquely at early, 88 at both early and late stage) and 546 were downregulated (500 only at early, 46 at both early and late stage). At late infection stage (72 hpi), the number of DEGs decreased significantly, with 282 upregulated (194 unique at late stage) and 395 downregulated (349 unique at late stage) genes (Fig 3A–3C). In case of C306, response at early infection stage was characterized by, upregulation of 416 genes (339 unique at early, 77 at early and late stages), which was relatively lower than HW2004, while downregulation was observed for 615 (600 uniquely at early, 15 at early and late stages) genes. At late stage (72 hpi) the number of DEGs decreased with 249 upregulated and 426 downregulated (Fig 3D–3F).

**Fig 4:**
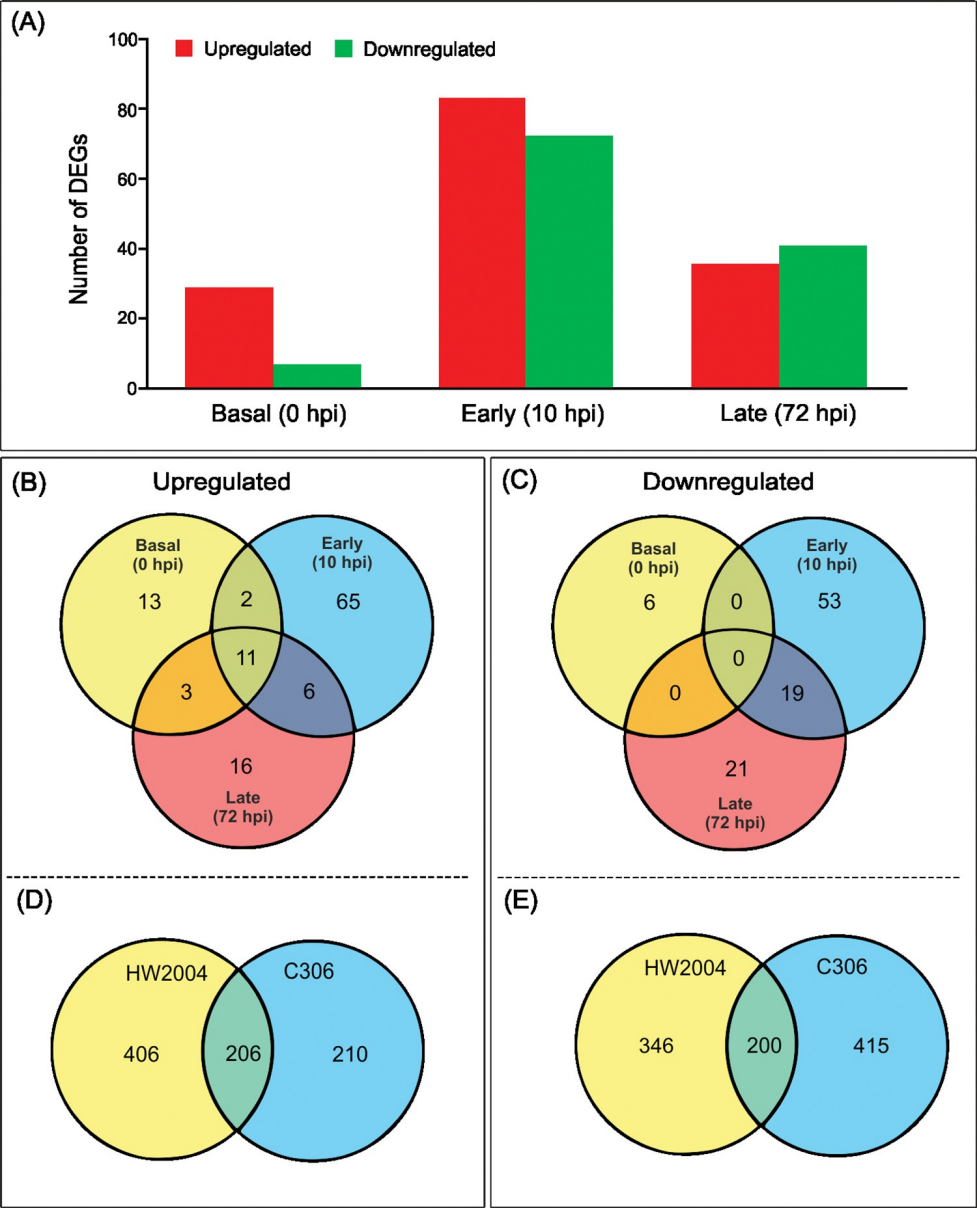
Comparative transcriptomics of wheat NILs for *Sr24*. (A) Number of DEGs in HW2004 compared to C306 at three stages of infection (0, 10 and 72 hpi). Venn diagram representation of number of unique and shared DEGs at different stages of infection: (B) Upregulated and (C) Downregulated genes in HW2004 at basal, early and late stages of infection. Common and unique DEGs among the two NILs at early (10 hpi) stage of infection: (D) upregulated and (E) downregulated genes. Numbers in the Venn diagram represent number of DEGs in the respective category.

High genetic similarity between the NILs C306 and HW2004 was evident in the minor transcriptomic differences at basal levels, with only 29 genes with higher and seven with lower transcript levels in HW2004 (Fig 4A). At early stage of infection (10 hpi), upregulation of 84 DEGs was observed specifically in HW2004, while 73 showed downregulation (Fig 4A). At 72 hpi number of DEGs were substantially reduced (36 upregulated, 41 downregulated) (Fig 4A). Further comparative analysis revealed that in HW2004, 11 DEGs were upregulated at basal, early and late stages, while 65 DEGs were uniquely upregulated at early stage (Fig 4B). On the contrary, there were no common downregulated DEGs across the three stages, however, 53 DEGs were downregulated at early stage, of which 19 remained downregulated even at later stages (Fig 4C). Among the early upregulated genes, 406 were specific to HW2004, while 206 were also upregulated in C306 (Fig 4D). For the early downregulated genes, 415 were downregulated only in C306, while 200 were also downregulated in HW2004 (Fig 4E). In general, HW2004 showed early induction of multiple defence response related genes compared to C306 (Tables 1 and 2).

**Table 1:** Functional annotation of representative differentially expressed genes in HW2004 at early infection stage.

TC No	Transcript ID	Uniprot ID	Annotation^$^	Fold change	Corrected *p*-value
**Upregulated transcripts**					
TC447588	TraesCS6D02G009600.1	A0A3B6Q8L7	Sugar efflux transporter	9.61	0.045
TC384101	TraesCS4D02G342800.1	A0A3B6JR64	Pore-forming toxin-like protein	8.94	0.049
TC389076	TraesCS2D02G033500.1	Q41522	Thiol protease	8.91	0.035
TC445367	TraesCS7A02G178300.1	A0A3B6RBG0	Cupin	8.85	0.028
TC422716	TraesCS4D02G021400.1	A0A3B6JDL9	Phosphomethylpyrimidine synthase	8.40	0.033
TC414244	TraesCS1A02G041400.1	A0A3B5XTU1	Potato inhibitor I family	7.32	0.036
TC381404	TraesCS1A02G335300.1	A0A3B5Y4N2	Caffeate O-methyltransferase	7.24	0.035
TC382069	TraesCS2A02G440300.1	A0A3B6B5L6	Plant lipid transfer protein	6.88	0.036
TC431012	TraesCS2B02G066000.1	A0A3B6BYF5	2OG-Fe(II) oxygenase superfamily	6.28	0.033
TC396288	TraesCSU02G131900.1	A0A3B6UBA6	Nucleoside phosphatase	6.06	0.048
TC425850	TraesCS2D02G528500.1	A0A3B6DM57	Lipoxygenase	6.05	0.041
TC405983	TraesCS7D02G333800.1	A0A3B6TGM2	Heavy-metal-associated domain	5.90	0.048
**Downregulated transcripts**					
TC381292	TraesCS7B02G188000.3	A0A3B6SH23	HTH myb-type domain protein	-8.81	0.030
TC421852	TraesCS7A02G299400.2	A0A3B6RFW9	HTH myb-type domain protein	-8.57	0.040
TC380047	TraesCS5D02G323400.1	A0A3B6MVM1	B-Box-type zinc finger	-6.56	0.002
TC388671	TraesCS1B02G461600.1	A0A3B5Z6C4	Major sigma-70 factor signature	-6.26	0.004
TC376216	TraesCS5B02G150600.1	A0A3B6LKB8	Nodulin-like	-6.22	0.007
TC381429	TraesCS2A02G100800.1	A0A3B6ATE0	Dof-type domain-containing protein	-5.88	0.045
TC374919	TraesCS2A02G393900.1	A0A3B6B235	Pentatricopeptide (PPR) repeat	-5.27	0.004
TC391255	TraesCS4B02G107200.1	A0A3B6INJ2	Pyruvate kinase (EC 2.7.1.40)	-5.13	0.040
TC389072	TraesCS2B02G491700.2	A0A3B6CD80	Carbohydrate binding domain	-4.76	0.016
TC371639	TraesCS3D02G127300.1	A0A3B6GN13	HCO3- transporter family	-4.60	0.021
BJ317431	TraesCS3B02G318600.2	A0A077S3N9	G protein beta WD-40 repeat	-4.26	0.004
TC400248	TraesCS2A02G214400.1	A0A3B6AVF8	GRAS domain family	-4.07	0.019

$ Based on Uniprot annotation

**Table 2:** Functional annotation of representative differentially expressed genes in HW2004 at late infection stage.

TC No	Transcript ID	Uniprot ID	Annotation^$^	Fold change	Corrected *p*-value
**Upregulated transcripts**					
TC458819	TraesCS2D02G339700.2	A0A3B6DFH2	BTB domain-containing protein	7.77	0.032
TC446015	TraesCS7D02G008700.1	Q8W430	Sucrose 1-fructosyltransferase	5.76	0.050
TC427838	TraesCS4A02G485400.2	Q8W431	Fructan 6-fructosyltransferase	4.68	0.000
TC419881	TraesCS6D02G326000.1	P69463	Cytochrome b6-f complex subunit 5	4.24	0.022
TC448584	TraesCS2D02G595100.1	A0A3B6DNB2	Nudix hydrolase domain protein	3.77	0.000
TC442996	TraesCS3D02G140800.1	A0A3B6GQI2	KOW domain-containing protein	3.76	0.036
TC406600	TraesCS1B02G124100.1	A0A3B5YUS5	Usp domain-containing protein	3.71	0.044
TC397659	TraesCS1A02G050600.1	W4ZVB1	Defensin	3.63	0.039
TC368948	TraesCS1B02G360800.1	A0A3B5Z358	Reticulon-like protein	3.51	0.036
TC379083	TraesCS3B02G039700.1	A0A077RRW5	Aspergillus nuclease	3.5	0.045
CV771045	TraesCS5D02G130000.1	W5FLI7	V-type proton ATPase proteolipid	3.49	0.036
TC444560	TraesCS7D02G417400.1	A0A3B6TWE0	Peroxidase (EC 1.11.1.7)	3.47	0.042
TC422014	TraesCS6B02G424600.2	A0A3B6PSN2	HTH myb-type domain protein	3.44	0.046
TC446611	TraesCS2D02G362000.3	A0A1D5UU66	Genome assembly, chromosome: II	3.37	0.039
TC427459	TraesCS2A02G133800.1	A0A3B6ASR0	Methyltransf_11 domain protein	3.34	0.022
TC422293	TraesCS4A02G389800.1	A0A3B6I097	Uncharacterized protein	3.31	0.039
TC426797	TraesCS7B02G450100.1	A0A3B6ST96	Senescence domain protein	3.24	0.049
TC409014	TraesCS7A02G424100.1	A0A3B6RP55	Peroxidase (EC 1.11.1.7)	3.2	0.041
TC378225	TraesCS3D02G356300.2	Q71CZ3	Multidrug resistance protein 2	3.1	0.028
CA640141	N/A	N/A	N/A	3.06	0.028
TC437104	TraesCS2A02G350700.1	A0A3B6B2F0	Chitinase (EC 3.2.1.14)	3.02	0.046
CA615546	TraesCS2A02G502400.2	A0A3B6B6C6	Plasma membrane ATPase	2.88	0.046
**Downregulated transcripts**					
TC455696	TraesCS2B02G272900.1	A0A3B6C5N0	Photosystem II 10 kDa polypeptide	-4.7	0.049
TC412373	TraesCSU02G105100.1	A0A341ZD84	Protein kinase domain protein	-4.29	0.049
TC391353	TraesCSU02G008100.1	A0A3B6U2Z1	Uncharacterized protein	-4.04	0.049
TC414877	TraesCS6D02G253100.1	A0A3B6QH15	RING-type E3 ubiquitin transferase	-4.03	0.049
TC458764	TraesCS6A02G107700.2	A0A3B6NMH1	UDP-N-acetylglucosamine	-3.78	0.049
TC419972	TraesCS5A02G380900.1	A0A3B6KPD3	BHLH domain-containing protein	-3.75	0.049
CA692579	TraesCS5D02G390500.1	A0A3B6MXM6	Uncharacterized protein	-3.71	0.049
TC412692	TraesCS7D02G386600.1	A0A3B6TVE5	Uncharacterized protein	-3.61	0.049
TC429715	TraesCS5B02G384500.1	A0A3B6LT05	BHLH domain-containing protein	-3.53	0.049
TC436920	TraesCS2A02G199700.1	A0A3B6AWC1	Uncharacterized protein	-3.52	0.049
TC390202	TraesCS5D02G137000.1	A0A3B6MMK4	Uncharacterized protein	-3.48	0.049

^$^ Based on Uniprot annotation; ^N/A^ Gene ID not retrievable

### HW2004 showed early pathogen detection and hypersensitive response

#### Early stage

**Fig 5:**
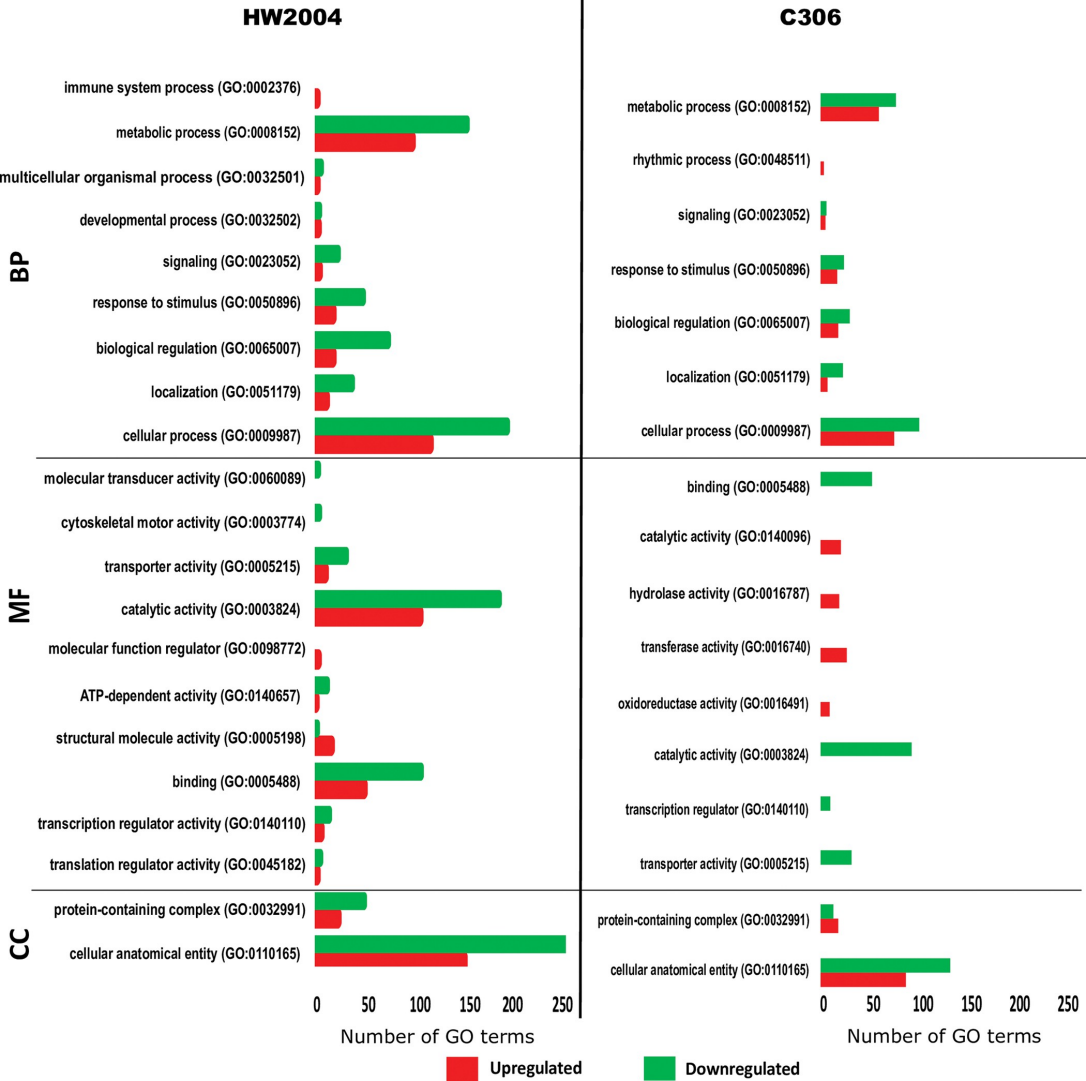
Gene Ontology (GO) characterization of upregulated genes. Overview of GO terms in (A) HW2004 and (B) C306. BP refers to Biological Process, MF to Molecular Function and CC to Cellular Compartment. The GO IDs of the respective GO terms are indicated in the parenthesis.

**Fig 6:**
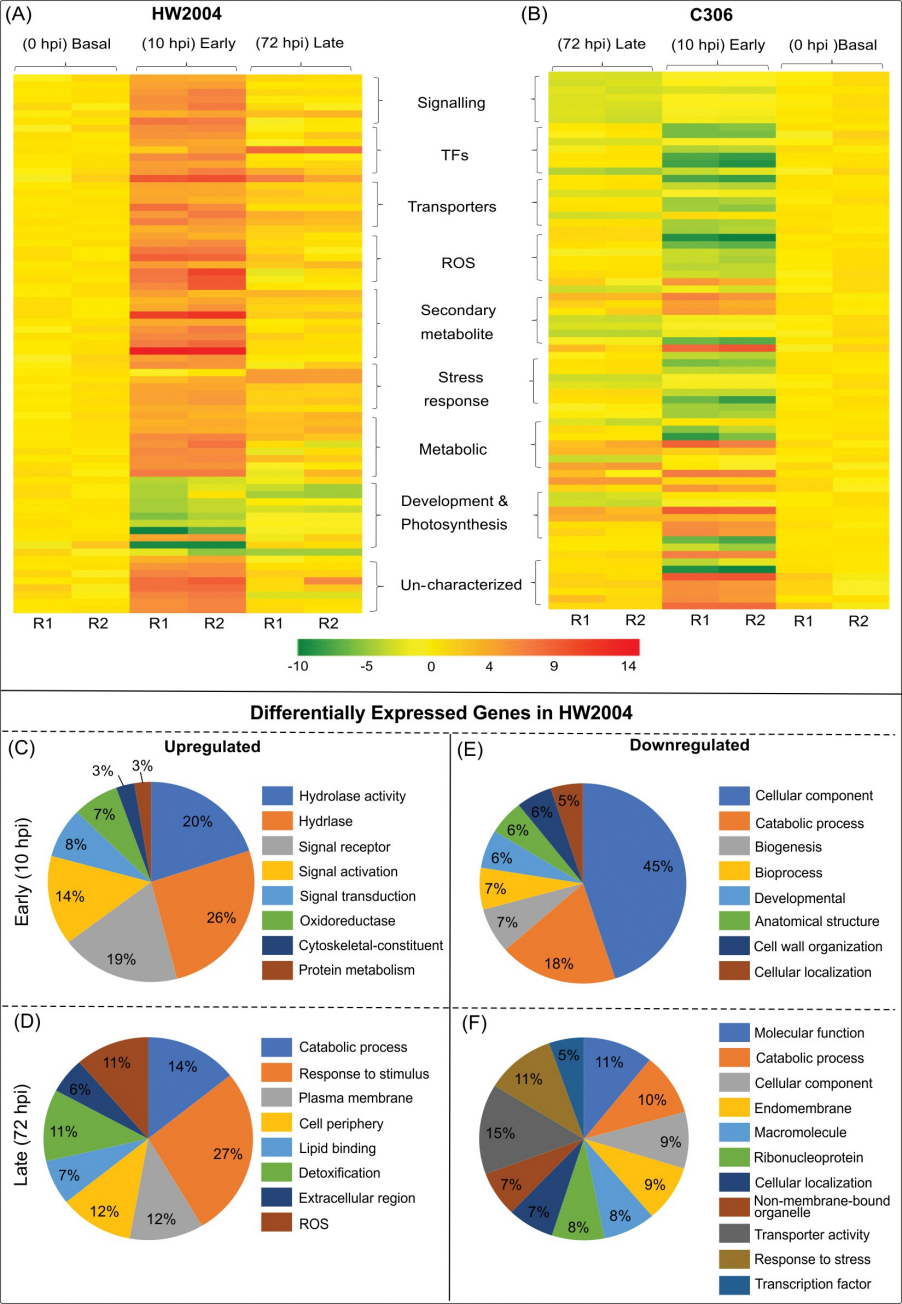
Heatmap representation for temporal expression pattern of representative disease response genes. Heat map profiles of several categories of stem rust responsive genes in (A) HW2004 and (B) C306. Labels in the middle represent broad biological function of the DEGs and colour scale represent the log_2_ fold-change of DEGs. R1 and R2 refer to the two independent biological replicates. Important biological categories of upregulated genes in HW2004 upon *Pgt* infection: (C) at early and (D) late stage, and downregulated genes at (E) early and (F) late stages.

In HW2004 peaking of transcriptomic response at early stage suggests that pathogen recognition and activation of defence response is an early event in wheat *Pgt* interaction. Among the upregulated DEGs important candidates included pathogen receptors and defence related TFs viz. NBS-LRR, Myb-like DNA-binding domain containing proteins. Genes involved in HR and neutralizing pathogen including SGNH hydrolase-type esterase, cytochrome P450, sugar efflux transporter, dirigent protein, phosphomethylpyrimidine synthase, Caffeate O-methyltransferase (COMT), 2OG-Fe(II) oxygenase, lipoxygenase were also upregulated (Table 1, Figs 5, 6A–6F, S2 Table). Further gene-based clustering and pathway analysis showed, that the upregulated genes were associated with signalling, response to stress, transporter activity, TF binding activity, cytoskeletal reorganization, HR mediating mechanisms i.e. ROS modulation, secondary metabolites, and lipid metabolism (Tables 3 and 4, S5 Fig). Thus, HW2004 due to presence of ‘*Sr24*’ seems to successfully detect *Pgt* and activate signalling mechanisms, which in turn activate defence related TFs to induce HR response against it.

**Table 3:** Biological function GO terms enriched in HW2004 in response to *Pgt*.

GO term	Biological function^#^	Number of hits	% of GO Terms	*p*-value
**Upregulated DEGs at early (10 hpi) stage of infection**				
GO:0005200	Structural constituent of cytoskeleton	75	6.38	0.00
GO:0005576	Extracellular region	3210	14.89	0.00
GO:0000226	Microtubule cytoskeleton organization	444	6.38	0.01
GO:0005198	Structural molecule activity	1760	8.51	0.02
GO:0003924	GTPase activity	782	6.38	0.03
GO:0007017	Microtubule-based process	759	6.38	0.03
GO:0005874	Microtubule	716	6.38	0.04
GO:0000278	Mitotic cell cycle	809	6.38	0.04
GO:0007010	Cytoskeleton organization	818	6.38	0.04
GO:0099081	Supramolecular polymer	731	6.38	0.04
GO:0099513	Polymeric cytoskeletal fiber	731	6.38	0.04
GO:0099080	Supramolecular complex	731	6.38	0.04
GO:0099512	Supramolecular fiber	731	6.38	0.04
GO:0009834	Plant-type secondary cell wall biogenesis	120	4.26	0.04
GO:0005525	GTP binding	997	6.38	0.05
GO:0032561	Guanyl ribonucleotide binding	997	6.38	0.05
GO:0001883	Purine nucleoside binding	997	6.38	0.05
GO:0032550	Purine ribonucleoside binding	997	6.38	0.05
GO:0015630	Microtubule cytoskeleton	819	6.38	0.05
GO:0019001	Guanyl nucleotide binding	1016	6.38	0.05
**Upregulated DEGs at late (72 hpi) stage of infection**				
GO:0005975	Carbohydrate metabolic process	4046	60.0	0.00
GO:0050738	Fructosyltransferase activity	2	20.0	0.00
GO:0004553	Hydrolyzing O-glycosyl compounds	1984	40.0	0.00
GO:0016798	Acting on glycosyl bonds	2266	40.0	0.00
GO:0004575	Sucrose alpha-glucosidase activity	63	20.0	0.01
GO:0004564	Beta-fructofuranosidase activity	65	20.0	0.01
GO:0090599	Alpha-glucosidase activity	88	20.0	0.01
GO:0015926	Glucosidase activity	465	20.0	0.05

# Based on GO annotation

**Table 4:** Pathways upregulated in HW2004 in response to *Pgt*.

Pathway ID	Pathway Description	Number of hits	% of genes	Genes involved
taes04145	Phagosome	268	6.38	TUBB5, TUBB3, TUBB2
taes01110	Biosynthesis of secondary metabolites	5240	10.64	LOC543072, TAAOS, LOC543365, CYS1, LOC543157
taes01100	Metabolic pathways	8808	10.64	LOC543072, TAAOS, LOC543365, CYS1, LOC543157
taes00500	Starch and sucrose metabolism	553	2.13	LOC543072
taes00950	Isoquinoline alkaloid biosynthesis	101	2.13	LOC543157
taes01230	Biosynthesis of amino acids	704	2.13	CYS1
taes00270	Cysteine and methionine metabolism	406	2.13	CYS1
taes00999	Biosynthesis of secondary metabolites	288	2.13	LOC543072
taes00350	Tyrosine metabolism	173	2.13	LOC543157
taes00920	Sulfur metabolism	117	2.13	CYS1
taes01200	Carbon metabolism	804	2.13	CYS1
taes03010	Ribosome	1041	2.13	LOC606335
taes00940	Phenylpropanoid biosynthesis	1041	2.13	LOC543365

The upregulation of signalling cascade at early stages of infection have also been reported in previous studies in case of wheat *Lr10* gene response, as well as other plant diseases [45–48]. Similar to current findings, *Lr28-* mediated resistance response has also shown enhanced cytoskeletal reorganization upon rust infection [49–51]. The HW2004 showed downregulation of genes related to chlorophyll a/b-binding protein, multiple Myb type TFs and zinc finger (B-Box type) containing protein (Table 1, S2 Table). In, addition some signalling genes (kinases, signal transduction) and TFs were also downregulated, suggesting that these might not be involved in defence response to *Pgt* in wheat (Figs 5 and 6).

#### Late stage

At late infection stage (72 hpi), the number of DEGs decreased significantly (Fig 3A–3C). The upregulated genes that showed sustained defence response, included genes for BTB domain containing protein, nudix hydrolase, glycosyl hydrolases, cytochrome b6-f complex subunit 5, defensin, nucleases and senescence domain containing protein (Table 2, S2 Table). GO annotation showed these genes to be related to signalling cascade, defence mechanism (NBS-LRR and ABC transporter protein), TFs, lipid metabolic, ROS burst, calcium flux modulating genes (EF hand calcium-binding domain profile protein), along with several unannotated genes (Table 2, Fig 6, S6 Fig, S2 Table). Carbohydrate metabolic pathways involved in multiple stress tolerance enhancing mechanisms were also upregulated at later stages (Table 4, S6 Fig).

At late infection stage downregulated genes included dirigent protein, agglutinin domain containing protein, germin-like protein, photosystem II related protein, reported to be downregulated in other rust diseases (Table 3, S2 Table) [50, 51]. These genes are primarily associated with basic growth and physiological processes and metabolic pathways (protein, carbohydrate, organic substance). This suggests that stem rust resistance response may also involve temporarily downregulation of growth and developmental pathways (S2 Table, S6 Fig).

### C306 showed impaired pathogen detection and defence response

#### Early stage

In case of susceptible genotype C306, response at early infection stage (10 hpi) was characterized by, upregulation of relatively lower number of genes than HW2004 (Fig 3D–3F). Lower number of upregulated genes in susceptible reaction, has been previously associated with faster growth and early establishment of pathogen [19, 52]. The downregulated genes included multiple hydrolases, Myb-type TFs involved in defence, proteins involved in detoxification (MATE efflux family protein), and candidates involved in pathogen sensing (serine/threonine kinases, NBS-LRR domain containing protein, serine/threonine protein kinases, major facilitator of sugar superfamily protein, MFS), suggesting an impaired pathogen detection, and absence of pathogen-specific defence responses (S3 Table). Although C306 also showed upregulation of some genes involved in signalling, transporters and ROS modulation, but the numbers were relatively less compared to HW2004, indicating lack of an effective HR. Additionally, it showed reduced restriction of its own reproductive and developmental processes (Fig 5, S6 Fig, S3 Table). Upregulated gene categories in C306 included germin like protein, dirigent protein, AAI domain containing protein, auxin-repressed protein, and genome assembly related protein (S3 Table, S6 Fig).

#### Late stage

At late stage (72 hpi) the downregulated genes included BHLH, PDZ6 and NBS-LRR domain-proteins, RING-type E3 ubiquitin ligase, Myb TF, glycosyl transferase and (S3 Table). As, these genes are associated with important defence related GO categories viz. response to biotic/abiotic stimuli and associated with downstream signalling, their downregulation is indicative of sustained failure of defence response (Fig 5, S3 Table). The upregulated genes consisted of few candidates central to plasma membrane localized stress responsive functions viz. signal receptor binding, kinases and transporters, but relatively lower in numbers and expression levels, indicating inadequate defence response to restrict pathogen growth. However, in contrast to HW2004, several metabolic, biosynthetic process related genes were upregulated in C306, thus showing reduced restriction of hosts own growth and developmental pathways (S6 Fig). It is very likely that some of these highly upregulated genes are responsible for susceptibility, by suppressing defence related genes [50, 51].

### Differential transcriptomic response in HW2004 and C306 upon *Pgt* infection

#### Wheat NILs showed DEGs at basal level

The two NILs showed minor differences at basal levels (Fig 4A). In the HW2004, basally, upregulated candidates included, GMC oxidoreductase, Hsp20, MFS type sugar transporter, SKP-1 like protein and an NBS-LRR, various R-proteins, transketolases and CED-4 domain containing protein (S4 Table). The NB-ARC domain and sugar transporter protein belong to ‘*R*’ gene family and involved in resistance to leaf and stripe rusts [5]. In addition, transketolase, oxidoreductase, and Hsp proteins are identified as key components of biotic stress response [53, 54]. Higher basal level of candidates, particularly those involved in pathogen detection, downstream signalling, defence mechanisms suggests enhanced levels of defence preparedness before pathogen infection, which may be central to “*R*” gene mediated resistance. Such basal expression differences of defence related genes has also been reported to contribute towards resistance to *Xanthomonas* infection in rice [48, 55]. Present study identified several unannotated genes with substantially higher basal levels in HW2004, viz. TC453923 (FC 8.33), TC443516 (FC 6.83), TC409011 (FC 6.59). These genes may serve as important candidates for further characterization to decipher their roles in stem rust resistance in wheat. The major downregulated DEGs included nuclear pre-mRNA splicing factor, 40S ribosomal protein, pectin acetyl esterase and some unknown genes. Few genes related to broad spectrum resistance viz. chitinase, peroxidase, displayed lower levels, indicating that these may not be involved at basal level and might be required at later stages (S4 Table).

#### HW2004 detects pathogen and activate HR at early stage

Comparative analysis at early stage of infection (10 hpi) revealed upregulation of candidates involved in pathogen recognition, defence signalling, TFs involved in activation of defence responsive genes in HW2004 (Fig 6, Table 5). Activation of HR, ABC transporters, PR proteins, glucosidases, fungal cell wall dissolving enzymes (pectin-glucuronyl transferase, glucanase, pectinase, hydrolases), ROS generation, and lipid metabolism genes was also observed (Fig 5, S4 Table). Similar involvement of early stage genes have been reported in wheat leaf and stripe rust response, in detection of effectors and activation of defence response [19, 50]. Early HR induction in presence of ‘*R*’ gene is a hallmark of specific defence response in plants [8]. The genes showing lower levels included a jasmonate induced protein, and multiple DEAD/DEAH box helicases, nuclear pre-splicing factor, and molecular chaperone SugE (Table 5, S4 Table). In general, pathways related to primary metabolism (carbohydrates, energy, amino acids, and lipids), and cellular process (processes related to cell growth, death and cellular community) were also downregulated (S7 Fig).

**Table 5:** Functional annotation of representative important differentially expressed genes in HW2004 compared to C306 at early infection stage.

TC No	Transcript ID	Uniprot ID	Annotation^$^	Fold change	*p*-value
**Upregulated transcripts**					
TC453923	N/A	N/A	N/A	9.28	2E-04
TC409011	TraesCS6B02G050600.1	A0A3B6PFF4	Transket_pyr domain-containing protein	9.08	0.009
TC430637	TraesCS1B02G481500.1	A0A3B5Z6W3	Uncharacterized protein	6.26	0.016
TC443516	N/A	N/A	N/A	6.08	0.002
TC423690	TraesCS2B02G310700.1	A0A3B6C716	Uncharacterized protein	5.81	0.018
TC405974	TraesCS5B02G015500.1	A0A3B6LFC8	Uncharacterized protein	5.75	0.042
TC390108	TraesCS1A02G165500.1	A0A3B5XYF4	Helitron_like_N domain protein	5.18	0.023
TC396071	TraesCS4A02G061900.1	A0A3B6HT09	Uncharacterized protein	4.86	0.016
TC423690	TraesCS2B02G310700.1	A0A3B6C716	Uncharacterized protein	4.82	0.031
CA618104	TraesCS5A02G355700.1	A0A3B6KP14	Uncharacterized protein	4.76	0.015
**Downregulated transcripts**					
TC409508	TraesCS3D02G544500.1	A0A3B6H7S0	Uncharacterized protein	-9.58	0.001
TC434139	TraesCS3D02G530700.1	A0A3B6H4K5	Transcription initiation factor IIA	-8.98	0.001
TC442680	TraesCS3D02G530600.2	A0A3B6H432	DEAD/DEAH box helicase	-8.6	0.000
TC431511	N/A	N/A	N/A	-7.94	0.000
CK151624	N/A	N/A	N/A	-7.75	0.002
TC441547	N/A	N/A	N/A	-7.58	0.002
TC417781	N/A	N/A	Prokaryotic membrane lipoprotein	-7.2	0.010
TC459502	TraesCS6B02G127000.1	A0A3B6PG83	HSF_DOMAIN domain protein	-6.75	0.001
TC405907	TraesCS3D02G537600.1	A0A3B6H3A6	Uncharacterized protein	-6.61	0.025
TC406137	TraesCS3D02G525300.1	A0A3B6H780	Diphosphomevalonate decarboxylase	-6.45	0.002
TC402750	TraesCS3D02G540200.1	A0A3B6H4E7	Uncharacterized protein	-6.21	0.003

^$^ Based on Uniprot annotation; ^N/A^ Gene ID not retrievable

#### Reduced but sustained defence response at late stages in HW2004

At 72 hpi number of DEGs were significantly reduced (Fig 4A–4C), which suggests that the modulation of crucial transcriptomic responses against stem rust pathogen occurs primarily at the early stages of infection. Findings in rice have also shown an early transcriptomic peaking response upon pathogen attack followed by a coordinated modulation of gene expression at later stages [18, 48]. Important upregulated genes included transket_pyr, BTB and Skp1 domain-containing protein, Mob1/phocein family protein (S4 Table). The upregulated genes were found to affect pathways related to genetic and environmental information processing, metabolism of lipids, terpenoids and polyketides, along with few involved in carbohydrate and protein metabolic pathways (S7 Fig). DEGs modulated at late stages of infection (2 to 5 dpi) are postulated to be associated with defence related pathways including HR, phytohormones mediated defence pathways, cell wall fortification mechanism, lipid peroxidation and modulation of carbohydrate metabolism [18, 47]. Also, levels of some of the immediate stress responsive GO groups viz. response to toxic substance, anchoring junction, ROS burst (oxidative stress response, H_2_O_2_ catabolism, and detoxification) were relatively reduced, indicative of their importance in the early infection stage (Figs 5 and 6).

**Fig 7:**
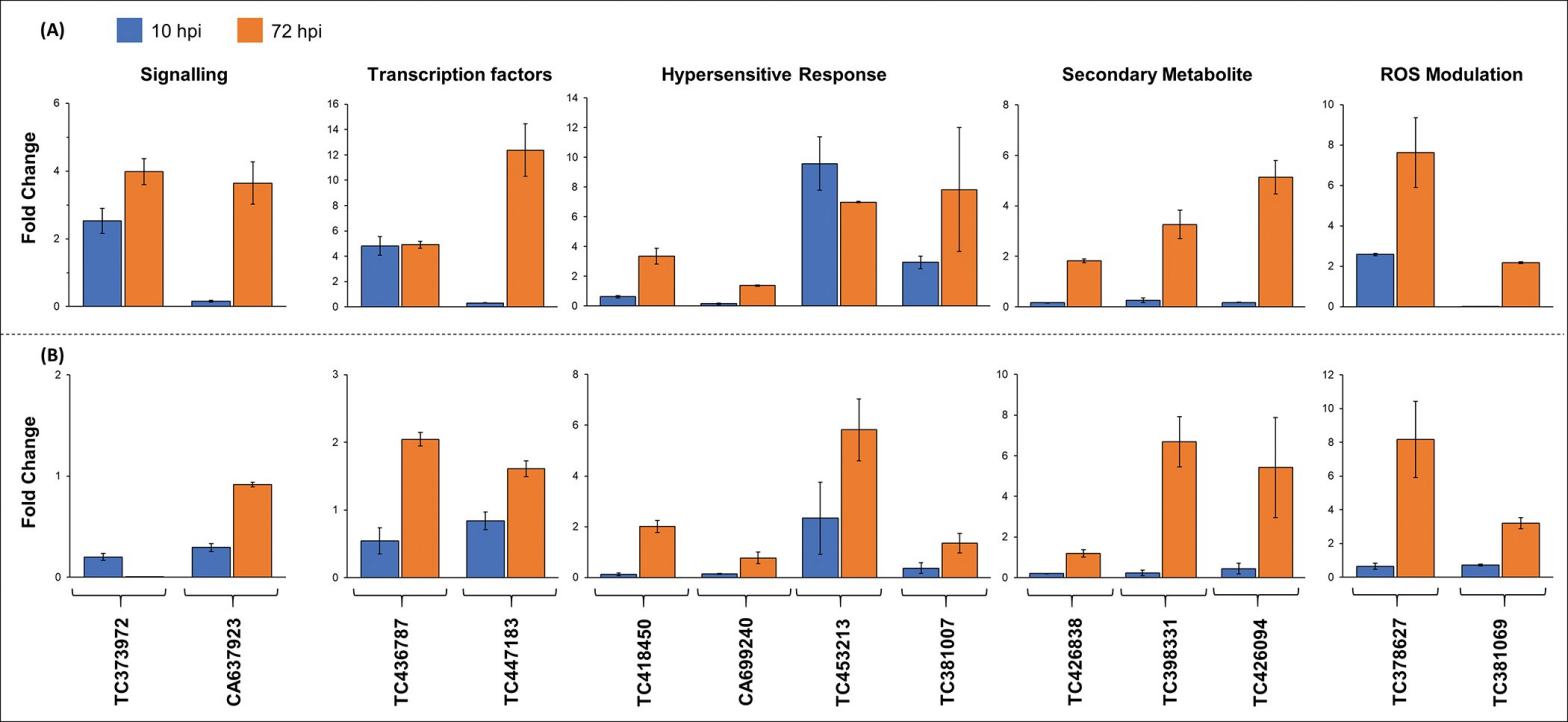
Expression pattern of representative DEGs, belonging to important defence responsive biological categories by RT-qPCR. Transcript response of key stem rust responsive genes at early (10 hpi) and late (72 hpi) stages of infection in top panel (A) for HW2004 and bottom panel (B) for C306. The functional categories in indicated on the top and the transcript IDs are given on the bottom side (TC373972: LRR containing kinase, CA637923: receptor protein kinase—like protein, TC436787: BHLH family protein-like, TC447183: WRKY45, TC418450: PR4, CA699240: β-1,3-glucanase, TC453213: Cytochrome P450, TC381007: Antifungal zeamatin-like protein, TC426838: Acetone-cyanohydrin lyase, TC398331: Caffeic acid O-methyltransferase, TC426094: Chalcone synthase, TC378627: Peroxidase-2, TC381069: α-ketoglutarate dehydrogenase). Fold changes were normalized to basal (0 hpi) stage. Error bars indicate SD of three independent biological replicates.

Expression profile by RT-qPCR of representative genes from different categories (defence, hypersensitive response, primary and secondary metabolism, unannotated) showed that their trend was consistent with microarray data (S8 Fig). RT-qPCR analysis of important candidates was carried out in both the NILs (Fig 7A and 7B), The HW2004 displayed upregulation of key signalling genes (kinases and NBS-LRR domain containing proteins) and TFs (WRKY45) involved in activation of defence responses (at early and late stages), while levels of these were lower in C306. Further, HW2004 also showed upregulation of genes associated with hypersensitive response (PR proteins, β-1,3-glucanase, Cytochrome P450), secondary metabolites (Caffeic acid O-methyltransferase, Chalcone synthase) and ROS modulation (peroxidases) compared to C306 that showed relative lower levels these genes, except those involved in some secondary metabolite synthesis and ROS modulation were upregulated, but only at later stages (Fig 7A and 7B).

#### Important components of resistance against stem rust of wheat

Differential expression analysis of wheat NILs upon *Pgt* infection, identified several key aspects mediating stem rust resistance response, including pathogen detection (signal receptor and transducing), activation of defence TFs and downstream induction of multiple defence processes, collectively helping in restricting pathogen growth.

### Signalling

Pathogen detection and signalling genes are crucial for early detection of infection and activation of defence responses via a complex cascade of pathways [11]. Upregulation of key signalling genes viz. Ser/Thr kinases, MAP Kinases and NBS-LRR specifically in HW2004, might be important for activation of HR and other defence mechanism against stem rust. HW2004 showed upregulation of higher number of NBS-LRR genes compared to C306, a known ‘*R*’ signature (Fig 6, Tables 1 and 2, S2 Table). Other studies have also shown similar expression profiles (both upregulated and downregulated) of NBS-LRR genes upon pathogen infection [50, 51]. Additionally, calcium and phosphatidylinositol signalling systems were also upregulated in the HW2004, which was consistent with a similar regulatory trend reported in case of *Lr28* [50, 51]. Likewise, cyclin-dependent protein kinases, cysteine rich receptor like protein kinases, diacylglycerol kinase 5, have also been reported to be important in other wheat rust interaction studies [16, 47, 48, 56, 57].

### Transcription factors

Upon pathogen reception, signalling cascade induces TFs involved in activation of defence responses [13]. Upregulation of multiple biotic stress responsive TFs (bZIP-like, BTF3b-like, AP2/EREBP type, WRKY and Hd1-like) in HW2004 is indicative of their roles in defence response against *Pgt* pathogen (Fig 6, Tables 1 and 2, S2 Table). Previous reports have also shown involvement of these TFs in activation of NBS-LRR genes, ROS burst, detoxification pathways, salicylic acid (SA)-mediated defence response, secondary metabolites (flavonoids) production and PR protein synthesis [50, 51]. On the contrary, C306 displayed relatively weak response of these candidate genes upon infection with *Pgt*. Differential modulation of TFs viz. Myb2, bZIP, AP2, BHLH, MADS box, multiple WRKY (WRKY69, 70, 33, 40) in response to rust pathogen has also been reported in previous studies [16, 18, 56].

### HR pathways

Biotic stress induced TFs are involved in activation of defence pathways. Several key genes central to the HR during plant pathogen interaction (ABC transporters, Cytochrome P450, WIR1, Caffeic acid–O methyltransferase (COMT), heat shock proteins, chitinase, alcohol dehydrogenase, ankyrin repeat profile containing protein) were upregulated in the HW2004, in response to stem rust infection (Fig 6, Tables 1 and 2, S2 Table). Moreover, HW2004 also displayed upregulation of candidates involved in HR signalling (phosphoinositide specific phospholipase), biosynthesis of secondary metabolites, SA biosynthesis and Systemic Acquired Resistance (SAR) (PR1, PR4, defensin, isochorismate synthase, UDP-glucosyltransferase, methylesterase) [2, 6]. Expression analysis also indicate cross talk between basal- and race-specific defence responses, as seen in other wheat rust interaction studies [50].

### Transporters

HW2004 showed upregulation of multiple transporter proteins (ABC, MFS type, zinc/iron) and many proteins with transporter domain (Fig 6, Tables 1 and 2, S2 Table). Such changes can potentially alter the intracellular metabolite conditions and may contribute to resistance against the pathogen, as shown in case of sugar transporters and other transmembrane transporters [5, 58]. Resistance mechanism in *Lr34* and *Lr10* have showed upregulation of ABC, MATE efflux, pleiotropic drug transporters [16, 47], while upregulation of ion flux transporters (Ca^2+^, K^+^, H^+^) have been reported in resistance response to *Xanthomonas* in rice [48].

### ROS modulation

Defence mechanisms activate ROS generation to restrict the pathogens [5, 11], and genes involved in ROS modulation are involved in the response to fungal pathogen infection in wheat [16, 47, 48, 50, 51]. In the current study, significant upregulation of peroxidases (ascorbate, glutathione), catalases, thioredoxins, multiple superoxide dismutase, glutathione-S-transferase, alternative oxidases were observed in HW2004 (Fig 6, Tables 1 and 2, S2 Table). Enhanced ROS levels, during initial stages of infection (starting from initial haustoria formation) helps in restricting the pathogen growth, however, at later stages a dynamic balance of ROS generation and elimination is maintained by the host to efficiently encounter pathogen along with maintenance of its redox environment [52].

### Secondary metabolites

Secondary metabolites such as flavonoids (derived from phenylpropanoid metabolism) comprise an integral component of plant defence responses against pathogens [56]. Genes involved in secondary metabolite biosynthesis and xenobiotic biodegradation were consistently upregulated in HW2004 (Fig 6, Tables 1 and 2, S2 Table). The phenylpropanoid pathway is responsible for biosynthesis of antipathogen compounds (anthocyanins, lignin, and phytoalexins), and is known to be induced for rust resistance mediated by *Lr34* gene [16]. Phenylalanine ammonia lyase (PAL, involved in synthesis of SA) also mediates biosynthesis of lignin leading to lignification and strengthening of cell wall against pathogen invasion [52]. Lipoxygenases a key enzymes of lipid metabolism is also involved in Jasmonic acid (JA) based signalling for defence response [48].

### Metabolic pathways

Defence response to pathogen also involves modulation of primary metabolic pathways (catering for energy requirement), to restrict pathogen growth. Genes involved in steroid biosynthesis and, linoleic acid metabolism were upregulated in HW2004 (Fig 6, Tables 1 and 2, S2 Table). This was consistent with the previously reported defence response to *Xanthomonas* [48]. Overall, carbohydrate metabolism was also modulated in both resistant and susceptible NILs. HW2004 showed downregulated tricarboxylic acid (TCA) cycle and upregulated glycolysis, suggesting preference for faster energy production, also use of alternate carbohydrate source (galactose). Previous report on *Lr34* response have also shown modulation of TCA cycle and GABA shunt pathway, while, *Lr1* response study showed upregulation of glycolysis [16, 18].

### Uncharacterized genes

Several uncharacterized genes were substantially upregulated in the resistant wheat NIL HW2004, some of which are likely to be involved in resistance to stem rust, with possible roles in detection of pathogen, or defence responses (Fig 6, Tables 1 and 2, S2 Table). Further characterization of these candidate genes will provide a better insight into molecular basis of wheat stem rust interaction.

### Multiple DEGs map to the translocated fragment of *Sr24* donor species

**Fig 8:**
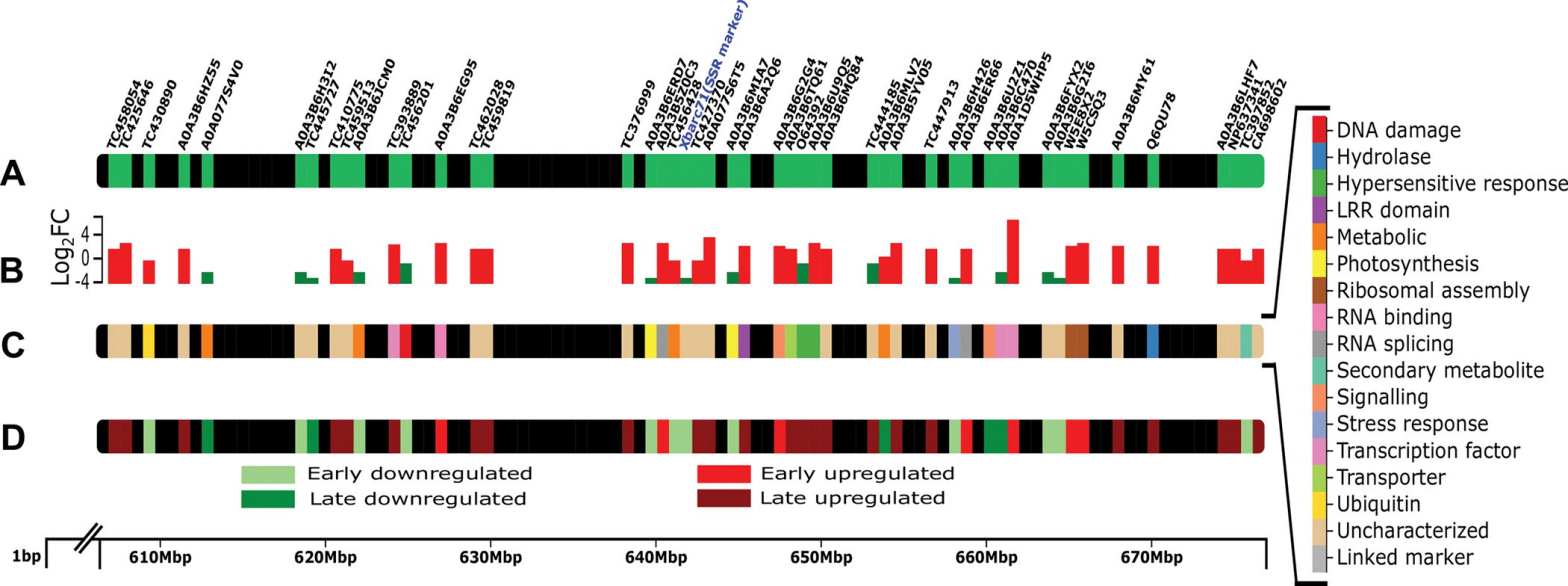
Characteristics of the DEGs in Wheat NIL HW2004 (post *Pgt* 7G11 infection) mapped on the 70 Mbp region of the translocated fragment (spanning the *Sr24*-linked marker, *Xbarc7*1) corresponding to the chromosome 3E of *Thinopyrum elongatum*. A) Relative positions of *Sr24*-linked marker *Xbarc71* and DEGs mapped across the 70 Mbp region of the translocated fragment. B) Relative expression levels (log_2_FC) of the DEGs. C) Biological functional categorization based on the Uniprot/TC ID information. D) Indication of post-infection time-course expression response (early/late and up/down) of the mapped DEGs. The scale on the bottom is indicative of the position of the DEGs in the 70 Mbp region, while the information about the functional categories (and colour codes) are indicated on the right-hand side panel.

The *Sr24* gene is introgressed in wheat due to a translocated fragment from *T*. *elongatum*. NCBI-Blast-based sequence similarity search mapped *77* DEGs with significant hits to the 70 Mbp region harbouring the *Sr24-*linked marker (*Xbarc-71* SSR marker) in *T*. *elongatum* (Fig 8). It is possible that these mapped transcripts may have originated from the *T*. *elongatum* fragment, and comprise some key genes involved in resistance mediated by *Sr24*. Of the upregulated DEGs, 10 were from the early stage, while 44 from late stage of infection (Table 6). These DEGs were related to biological functional category of signalling, LRR domain containing proteins, and TFs, suggesting their role in defence activation. In addition, some DEGs belonged to defence response categories viz. HR, Transporters and Hydrolases. These are potential candidates for primary transcriptional signal response upon *Pgt* infection, which further activates an elaborated host defence response. Among the downregulated DEGs 16 were from early stage and seven from later stage of infection which were primarily related to metabolic and photosynthetic roles (S5 Table). It is interesting to note that many of the uncharacterized/unannotated DEGs also mapped to *T*. *elongatum* translocated fragment and were close to the *Sr24-*linked SSR marker *Xbarc71* (Fig 8), some of which might be important for the rust resistance. The limited understanding of roles of the genes originating from these wild relatives of wheat, advocates their detailed characterization for exploitation of their hidden potential in disease resistance breeding [24].

**Table 6:** *Pgt* induced representative DEGs from HW2004 showing similarity to *Thinopyrum elongatum* chromosome 3E, in vicinity to region linked with marker of *Sr24*.

Gene ID	Start Coordinate	End Coordinate	Percent Identity	Query coverage	log_2_FC	Biological function^#^
**Early upregulated**						
TraesCS3D02G531900.1	661992238	661991624	84.34	71	6.75	Transcription factor
TraesCS2D02G362000.3	640629710	640629376	79.40	44	3.25	RNA splicing
TraesCS1B02G308600.1	640608736	640608470	93.70	61	3.12	Signalling
TraesCS3B02G602100.1	665823663	665823347	93.08	86	2.89	Ribosomal assembly
TraesCS3B02G581900.1	647477974	647478204	97.84	75	2.69	Signalling
TC456428*	641420105	641419790	93.08	50	2.68	Uncharacterized
TraesCS5D02G079000.1	654314385	654314604	83.64	48	2.67	Uncharacterized
TraesCS3A02G174900.1	627165162	627165473	96.15	76	2.62	RNA binding
TraesCS3A02G530600.1	665301076	665300730	85.96	94	2.42	Ribosomal assembly
TraesCS3A02G525400.1	658662233	658663012	95.78	99	2.34	RNA splicing
**Late upregulated**						
TraesCS3B02G578200.1	643287510	643288007	91.03	96	3.93	Uncharacterized
TraesCS1B02G308600.1	640608736	640608470	93.70	61	3.46	Signalling
TraesCS7B02G243000.1	670065110	670064575	75.23	48	3.22	Transporter
TraesCS3B02G602100.1	665823663	665823347	93.08	86	2.89	Ribosomal assembly
TraesCS1D02G449300.1	645177651	645177267	91.45	47	2.58	LRR domain
TraesCS1A02G127100.2	624163095	624161971	89.22	99	2.58	Transcription factor
TraesCSU02G011700.1	649535496	649536213	79.70	62	2.57	Hypersensitive response
TraesCS3A02G517100.1	649285716	649286273	90.00	61	2.08	Hypersensitive response
TraesCS7D02G238600.1	648182569	648185099	97.50	99	2.04	Transporter
**Early downregulated**						
TraesCS5D02G010000.1	644477884	644478322	94.81	53	-2.22	Photosynthesis
TraesCS3B02G538000.1	609222820	609223272	89.85	99	-2.32	Ubiquitin
TraesCS4D02G017900.2	622538169	622538490	91.93	77	-2.36	Metabolic
TraesCS3A02G506200.1	639666652	639667251	96.33	99	-2.58	Photosynthesis
TraesCS3D02G503700.1	625104970	625105993	97.85	99	-2.62	DNA damage
TraesCS3A02G525100.1	658540874	658540328	88.31	100	-2.85	Stress response
TraesCS1D02G181000.1	641032661	641032497	96.97	41	-2.98	Metabolic
TraesCS1D02G110800.1	641784803	641783949	83.35	98	-3.08	Uncharacterized
TraesCS3D02G530300.1	658542358	658542061	94.30	99	-3.38	Uncharacterized
**Linked Marker**						
*Xbarc71*	642313093	642312715				

^#^ Based on GO annotation

* Gene ID not retrievable

## Conclusion

**Fig 9:**
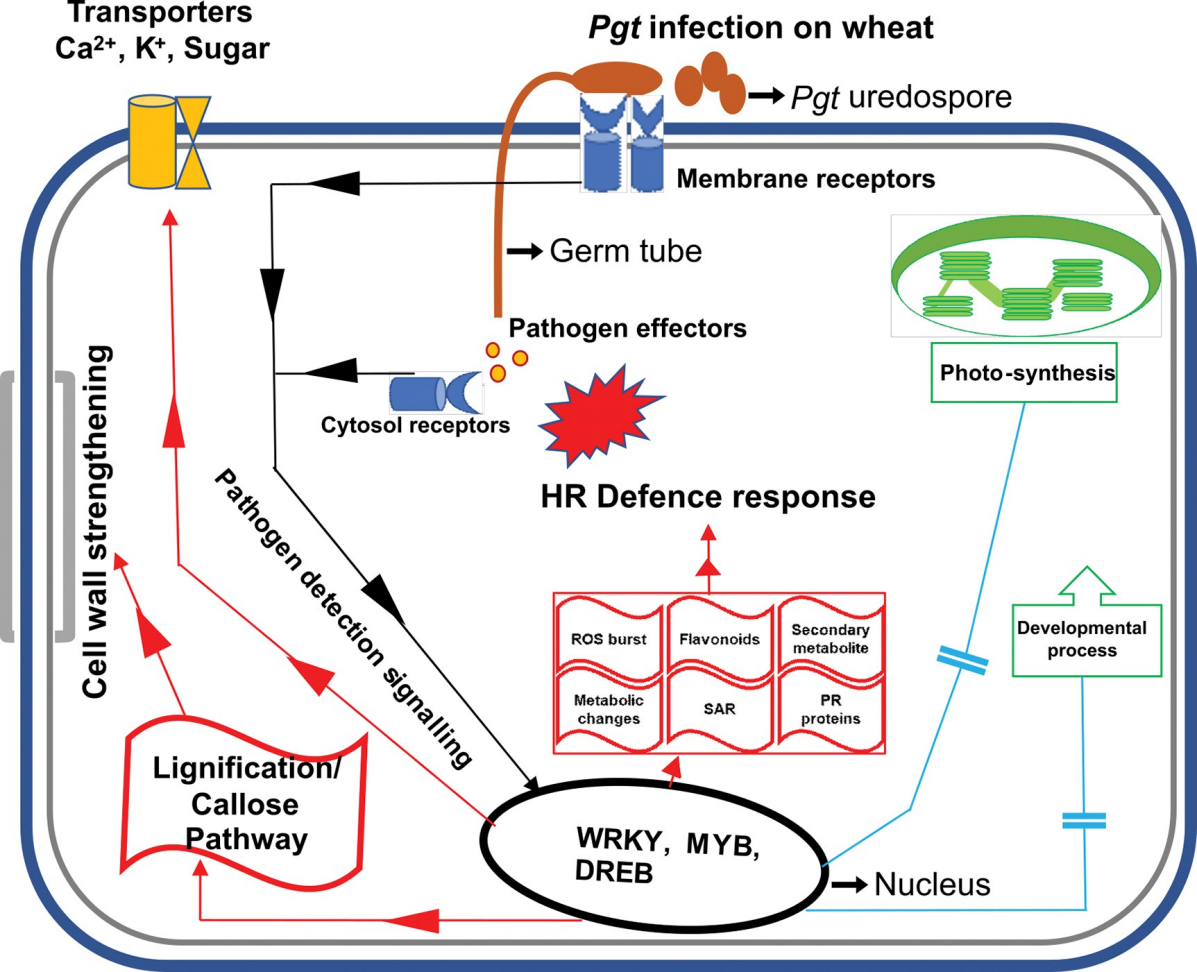
Schematic representation of probable mechanism of *Sr24*-mediated resistance in wheat upon *Pgt* infection. Black coloured lines represent sequence of events and candidates involved in pathogen detection and activation of defence responses. Red coloured lines and shapes represent upregulated pathways and processes upon *Pgt* infection, while blue coloured lines represent pathways which are repressed upon *Pgt* infection.

Current study utilized wheat NILs for stem rust resistance gene *Sr24*, for studying transcriptomics difference in response to *Pgt* race 7G11. Comparative transcriptomic analysis revealed higher basal levels of genes involved in defence to pathogen, in resistant NIL HW2004. HW2004 also showed early pathogen detection and defence responses with activation of plasma membrane associated receptors coupled with kinases, suggesting initiation of complex signalling cascades to activate both broad spectrum and specific defence responses. Study suggests that a combination of specific and basal defence response involving ROS generation, cell wall fortification, PR proteins, antifungal products of phenylpropanoid pathways and hydrolases, seems to be responsible for restricting the pathogen growth (Fig 9). Further, many highly over and under expressed uncharacterized genes were identified. These genes may be important in defence response to stem rust and may be characterized to decipher molecular nature and significance in future. Overall this study is helpful in understanding the molecular basis of defence response to stem rust in wheat.

## Supporting information

S1 Table List of oligonucleotide primers used for RT-qPCR analysis. (XLSX)

S2 Table List of DEGs identified at early and late infection stages in HW2004. (XLSX)

S3 Table List of DEGs identified at early and late infection stages in C306. (XLSX)

S4 Table List of DEGs identified at basal, early and late stage of infection in HW2004 compared to C306. (XLSX)

S5 Table List of *Pgt* induced DEGs from HW2004 at early and late stages of infection, with homology-based mapping to the selective region of *Thinopyrum elongatum* chromosome 3E. (XLSX)

S1 Fig Principal Component Analysis (in three-dimensional map) of the expression profiles of wheat *Pgt* infected leaf samples of HW2004 and C306. (TIF)

S2 Fig Volcano plot representation of differentially expressed genes in HW2004 and C306 after *Pgt* inoculation at three time points. Expression data of genes are plotted as log_2_ fold change versus -log_10_ FDR corrected *p*-value. Red dots represent significantly upregulated while green genes significantly downregulated DEGs respectively. * Denotes comparison of expression of DEGs at 72 hpi in HW2004 compared to 10 hpi.
(TIF)

S3 Fig Expression profile at early and late stages of infection upon *Pgt* infection. In HW2004 (A), C306 (B) based on hierarchical clustering. Global expression profile of differentially expressed genes in HW2004 compared to C306 (C).
(TIF)

S4 Fig Gene-based cluster analysis of DEGs in HW2004 and C306 after *Pgt* infection. (TIF)

S5 Fig Pathways affected in HW2004 at early stage of infection. Upregulated genes (A), downregulated genes (B).
(TIF)

S6 Fig Pathways affected in HW2004 at late stage of infection. By upregulated genes (A), downregulated genes (B), in C306 at early stage (C), (D) and at late stage of infection (E), (F).
(TIF)

S7 Fig Pathways affected in HW2004 compared to C306. By early stage upregulated (A), downregulated (B) and late stage upregulated (C), downregulated (D) genes.
(TIF)

S8 Fig Comparison of RT-qPCR-based validation of representative DEGs with their corresponding microarray expression pattern. In HW2004 compared to C306 at early stage (10 hpi) of infection. Error bars indicate SD of three independent biological replicates of RT-qPCR.
(TIF)

## Abbreviations

GO: Gene Ontology
PR: Pathogenesis Related
PAMP: Pathogen Associated Molecular Pattern, PRRs: Pattern Recognition Receptors
PTI: PAMP Triggered Immunity
LRR: Leucine Rich Repeats
ROS: Reactive Oxygen Species
ETI: Effector Triggered Immunity
HR: Hypersensitive Response
NBS-LRR: Nucleotide-Binding Site–Leucine Rich Repeats
SGNH: Serine (S), Glycine (G), Asparagine (N), and Histidine (H)
GMC: Glucose-Methanol-Choline
Hsp: Heat shock protein
SKP: S-phase Kinase-associated Protein
NB-ARC: Nucleotide-Binding domain shared with APAF-1, R proteins, and CED-4
CED: Cell Death protein
BTB: Bric-a-brac, Tramtrack Broad complex
ABC: ATP Binding Cassette
MATE: Multidrug And Toxic Compound Extrusion
Dof: DNA-binding with one finger
RING: Really Interesting New Gene
AAI: Alpha (α)-Amylase Inhibitor
PDZ: Post synaptic Density Protein
COMT: Caffeic acid O-Methyl Transferase
Mob: Mps1-binder-related
bZIP: Basic leucine Zipper
BTF3b: Basic Transcription Factor 3
AP2: Apetala2
EREBP: Ethylene-Responsive Element Binding Protein
Hd1: Heading date 1
BHLH: Basic Helix-Loop-Helix
MADS: M for minichromosome maintenance factor 1, A for Agamous, D for Deficiens, S for Serum Response Factor
