# Supplementary figures and images for: Comparative transcriptomics of stem rust resistance in wheat NILs mediated by Sr24 rust resistance gene
Source: PLoS One. 2023 Dec 11;18(12):e0295202. doi: 10.1371/journal.pone.0295202 (PMC10712884; doi:10.1371/journal.pone.0295202)

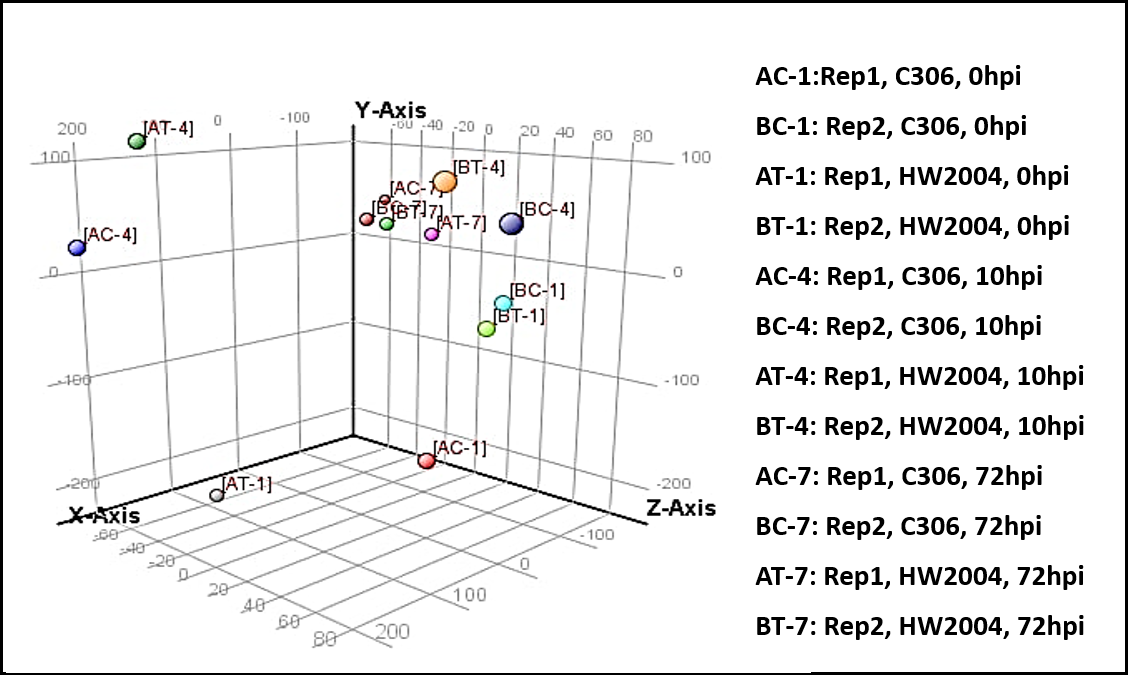

Supplement: S1 Fig — (TIF) [file pone.0295202.s006.tif]

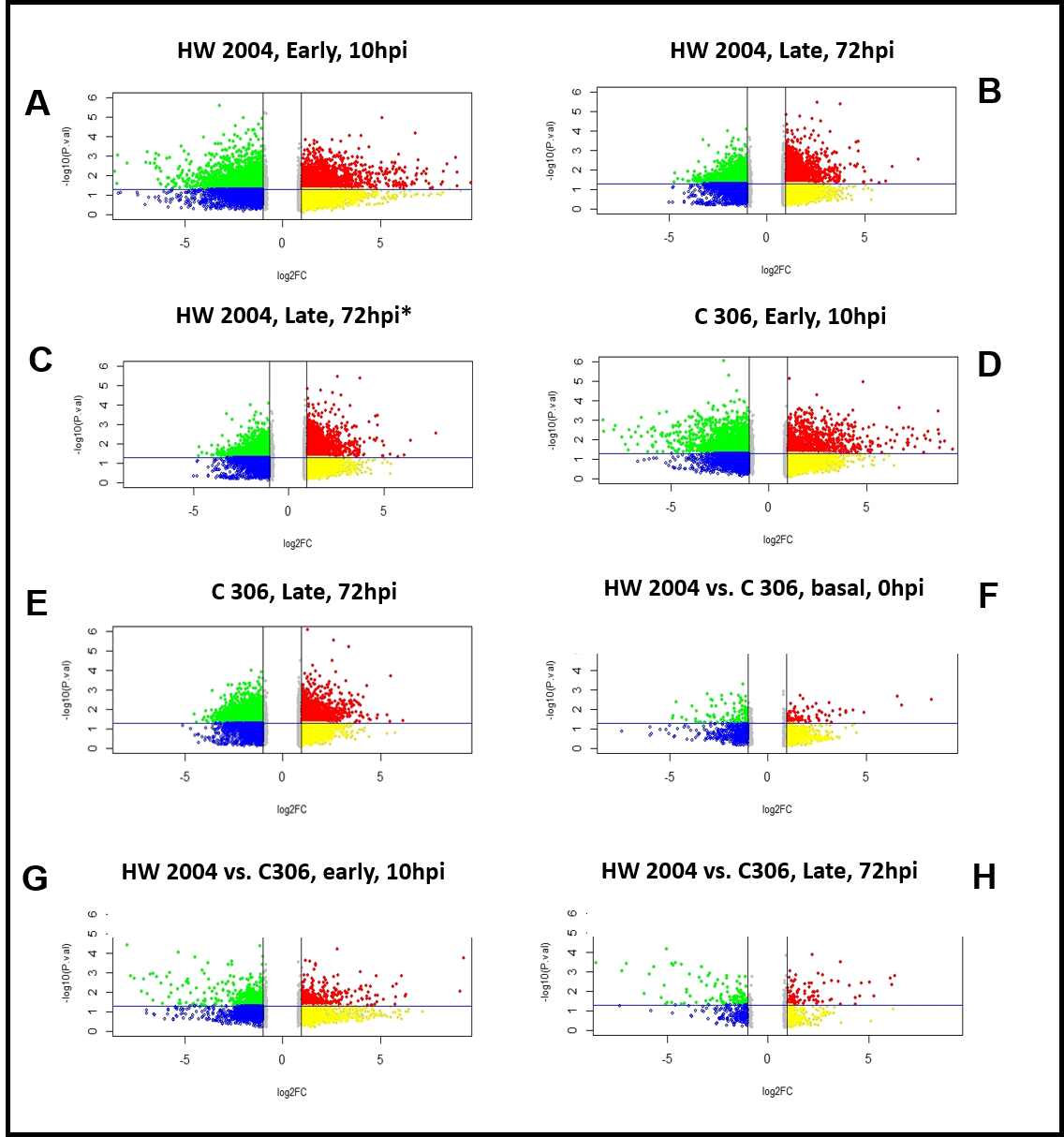

Supplement: S2 Fig — Expression data of genes are plotted as log2 fold change versus -log10 FDR corrected p-value. Red dots represent significantly upregulated while green genes significantly downregulated DEGs respectively. * Denotes comparison of expression of DEGs at 72 hpi in HW2004 compared to 10 hpi. (TIF) [file pone.0295202.s007.tif]

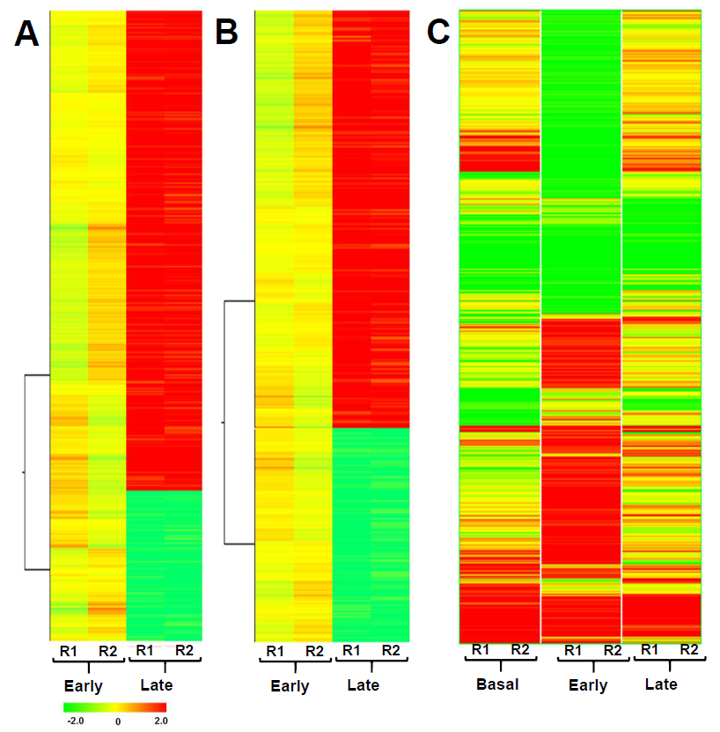

Supplement: S3 Fig — In HW2004 (A), C306 (B) based on hierarchical clustering. Global expression profile of differentially expressed genes in HW2004 compared to C306 (C). (TIF) [file pone.0295202.s008.tif]

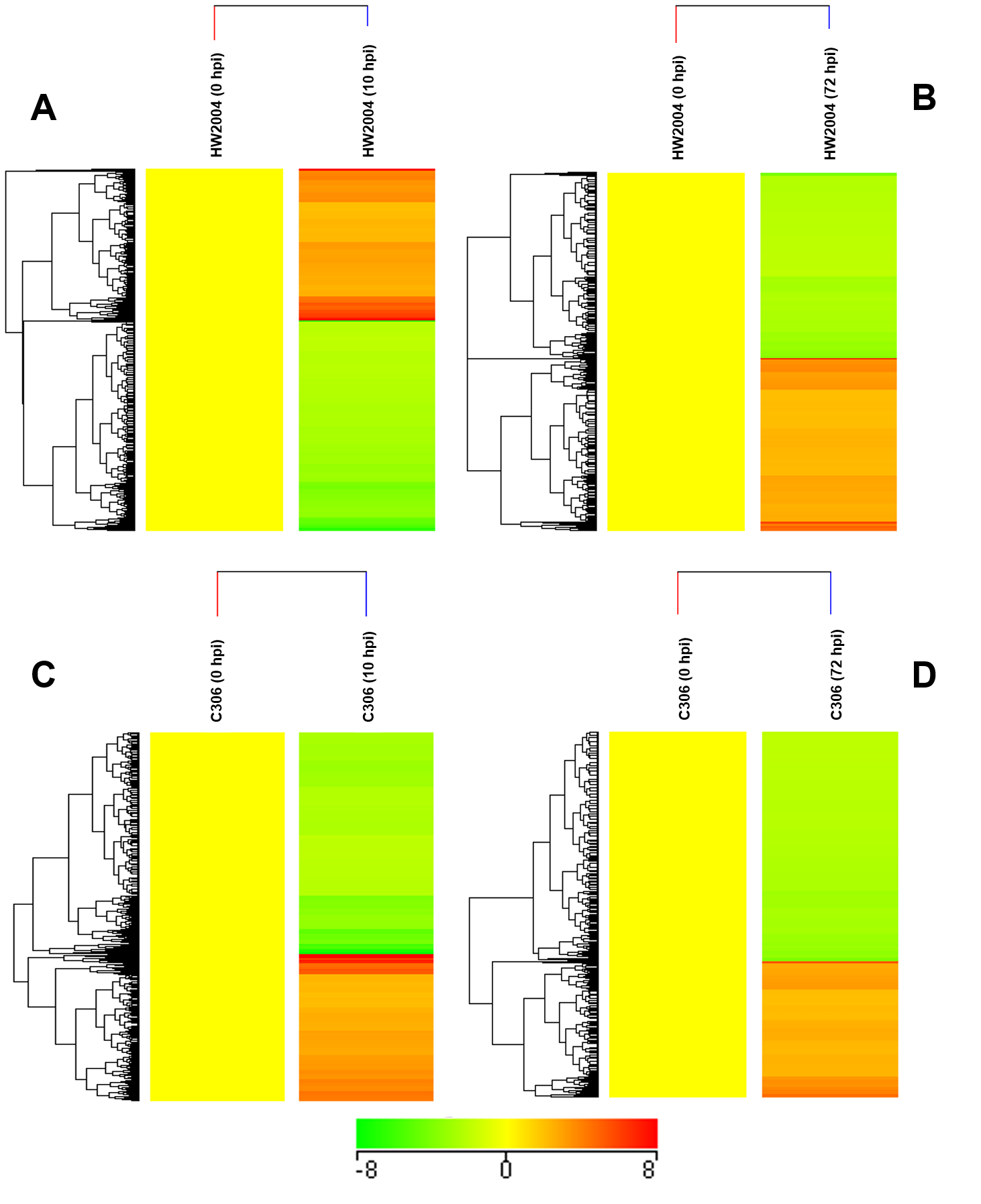

Supplement: S4 Fig — (TIF) [file pone.0295202.s009.tif]

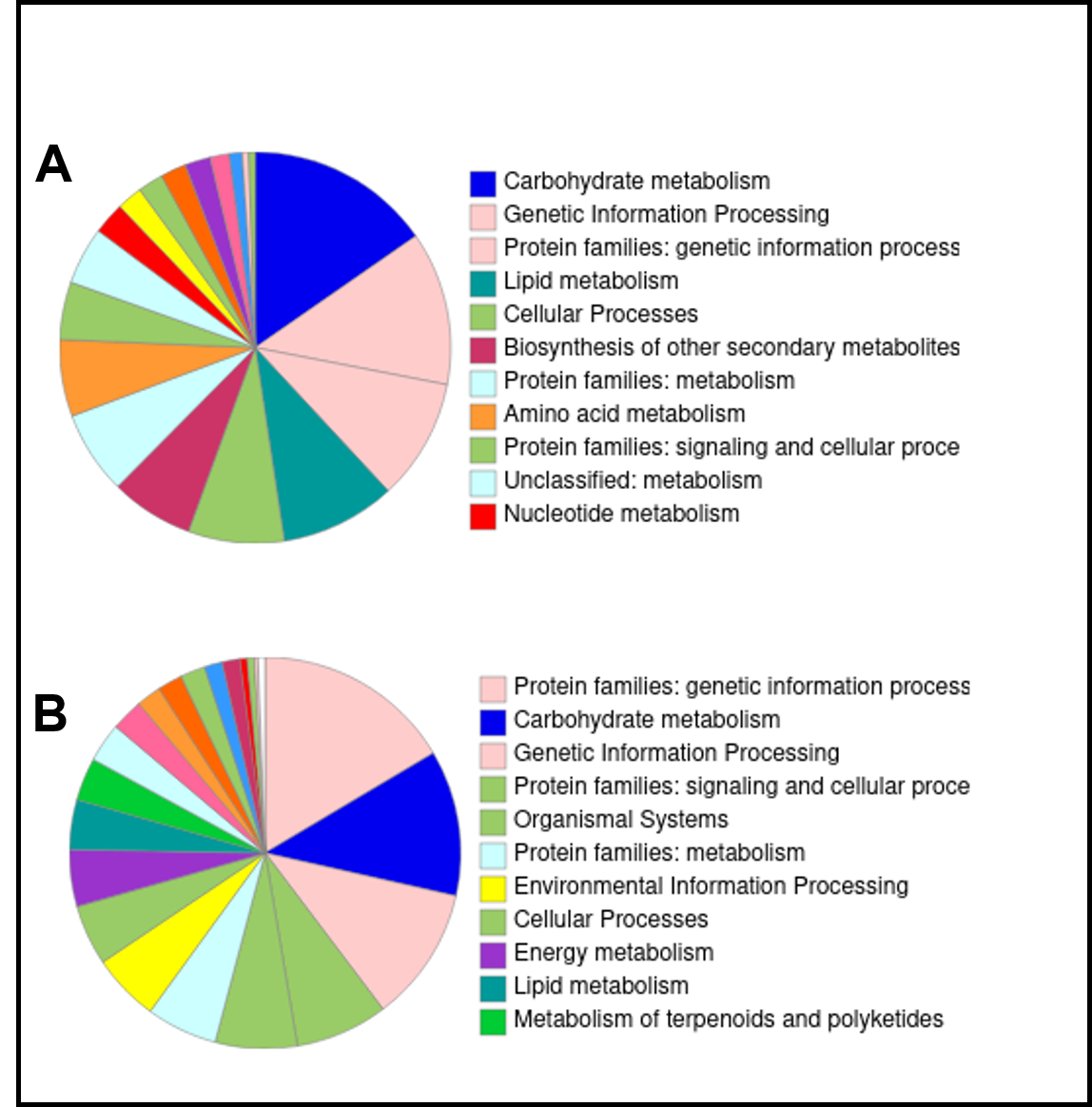

Supplement: S5 Fig — Upregulated genes (A), downregulated genes (B). (TIF) [file pone.0295202.s010.tif]

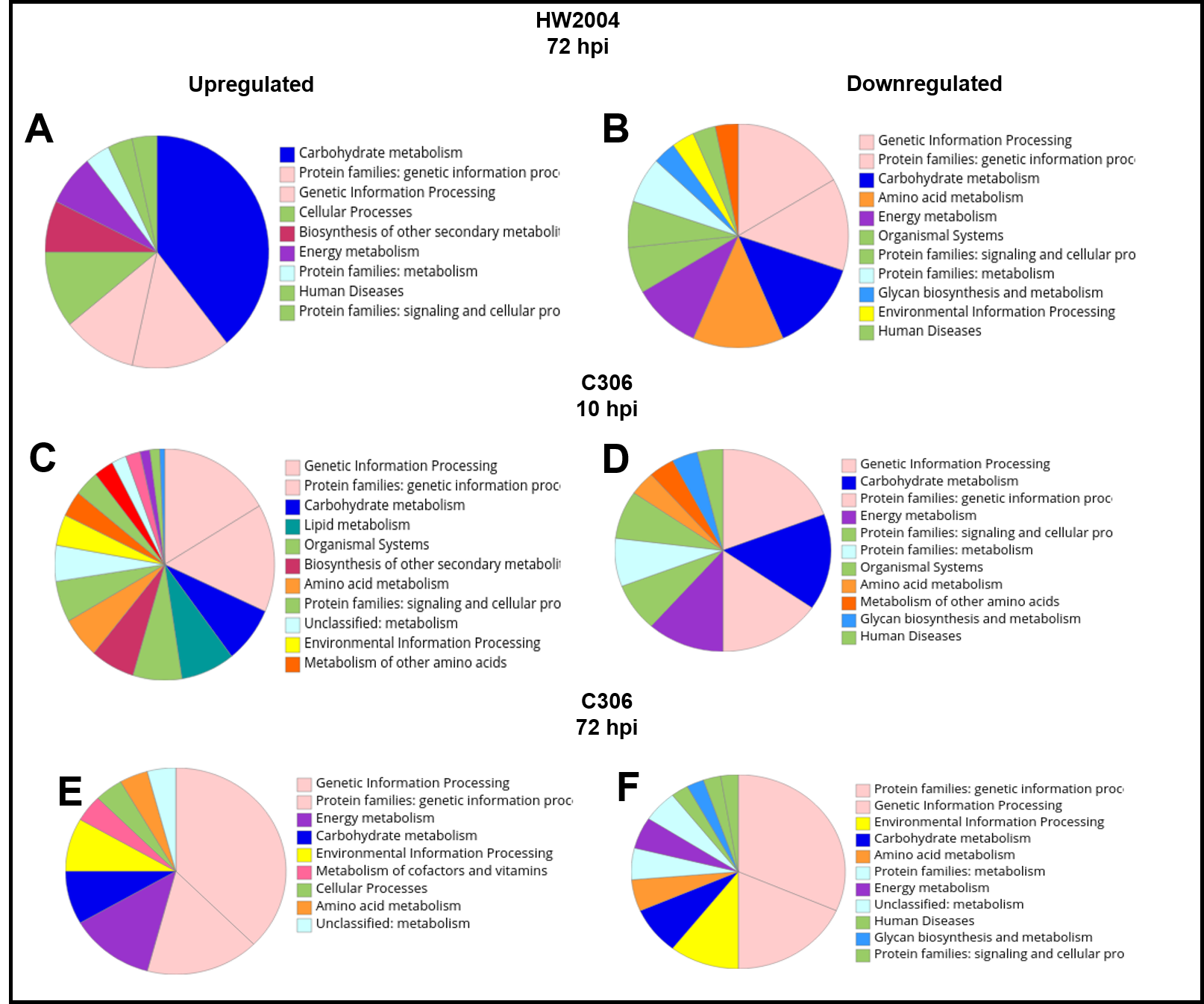

Supplement: S6 Fig — By upregulated genes (A), downregulated genes (B), in C306 at early stage (C), (D) and at late stage of infection (E), (F). (TIF) [file pone.0295202.s011.tif]

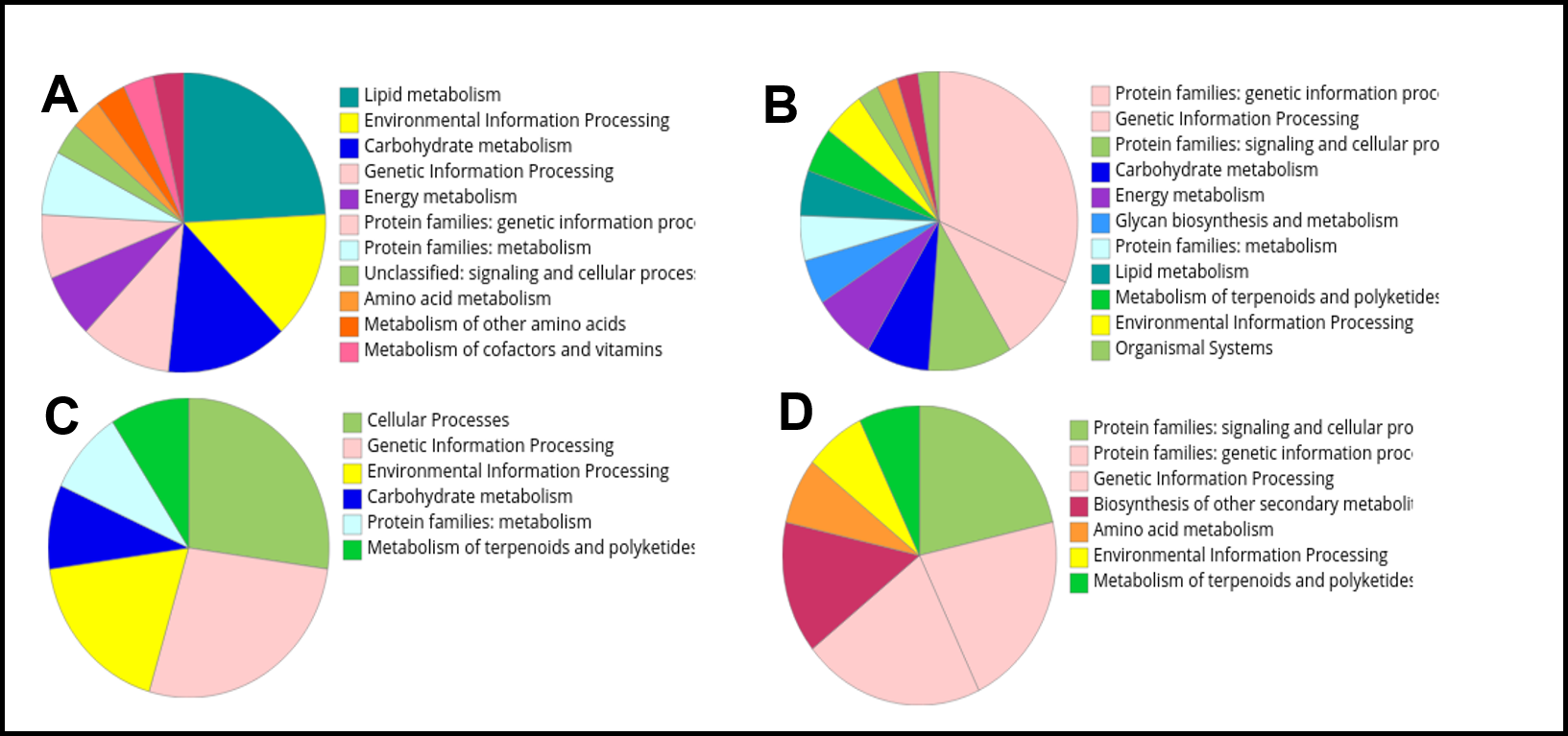

Supplement: S7 Fig — By early stage upregulated (A), downregulated (B) and late stage upregulated (C), downregulated (D) genes. (TIF) [file pone.0295202.s012.tif]

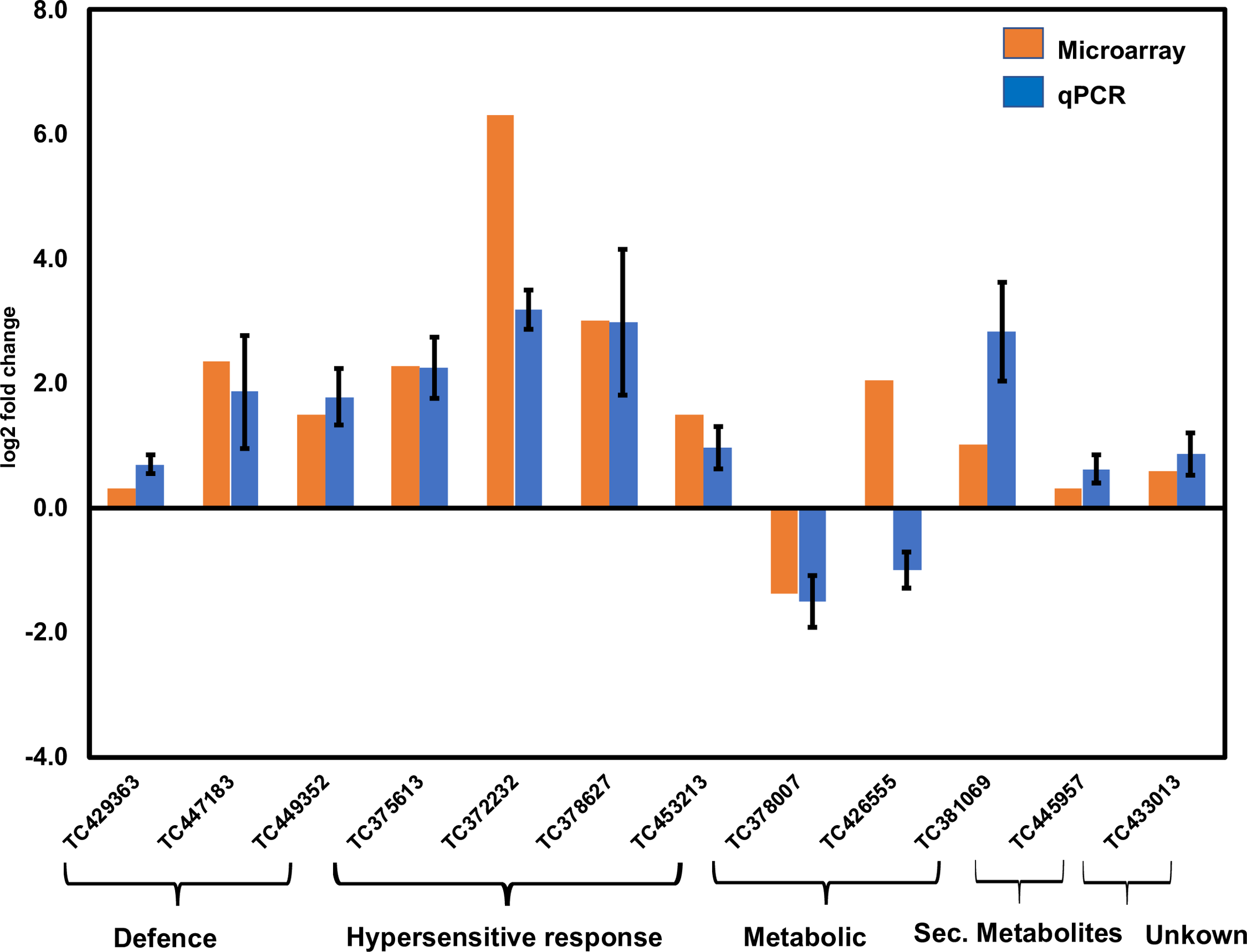

Supplement: S8 Fig — In HW2004 compared to C306 at early stage (10 hpi) of infection. Error bars indicate SD of three independent biological replicates of RT-qPCR. (TIF) [file pone.0295202.s013.tif]
